# In-Depth Characterization of microRNA Transcriptome in Melanoma

**DOI:** 10.1371/journal.pone.0072699

**Published:** 2013-09-04

**Authors:** James Kozubek, Zhihai Ma, Elizabeth Fleming, Tatiana Duggan, Rong Wu, Dong-Guk Shin, Soheil S. Dadras

**Affiliations:** 1 Department of Genetics and Developmental Biology, University of Connecticut Health Center, Farmington, Connecticut, United States of America; 2 Department of Computer Science and Engineering, University of Connecticut, Storrs, Connecticut, United States of America; 3 Department of Genetics, Stanford University School of Medicine, Stanford, California, United States of America; 4 Connecticut Institute for Clinical and Translational Science Biostatics Center, University of Connecticut Health Center, Farmington, Connecticut, United States of America; 5 Department of Dermatology, University of Connecticut Health Center, Farmington, Connecticut, United States of America; University of Tennessee, United States of America

## Abstract

The full repertoire of human microRNAs (miRNAs) that could distinguish common (benign) nevi from cutaneous (malignant) melanomas remains to be established. In an effort to gain further insight into the role of miRNAs in melanoma, we applied Illumina next-generation sequencing (NGS) platform to carry out an in-depth analysis of miRNA transcriptome in biopsies of nevi, thick primary (>4.0 mm) and metastatic melanomas with matched normal skin in parallel to melanocytes and melanoma cell lines (both primary and metastatic) (n = 28). From this data representing 698 known miRNAs, we defined a set of top-40 list, which properly classified normal from cancer; also confirming 23 (58%) previously discovered miRNAs while introducing an additional 17 (42%) known and top-15 putative novel candidate miRNAs deregulated during melanoma progression. Surprisingly, the miRNA signature distinguishing specimens of melanoma from nevus was significantly different than that of melanoma cell lines from melanocytes. Among the top list, miR-203, miR-204-5p, miR-205-5p, miR-211-5p, miR-23b-3p, miR-26a-5p and miR-26b-5p were decreased in melanomas vs. nevi. In a validation cohort (n = 101), we verified the NGS results by qRT-PCR and showed that receiver-operating characteristic curves for miR-211-5p expression accurately discriminated invasive melanoma (AUC = 0.933), melanoma in situ (AUC = 0.933) and dysplastic (atypical) nevi (AUC = 0.951) from common nevi. Target prediction analysis of co-transcribed miRNAs showed a cooperative regulation of key elements in the MAPK signaling pathway. Furthermore, we found extensive sequence variations (isomiRs) and other non-coding small RNAs revealing a complex melanoma transcriptome. Deep-sequencing small RNAs directly from clinically defined specimens provides a robust strategy to improve melanoma diagnostics.

## Introduction

The incidence and mortality of melanoma have continually increased over the past decades in the US. It is estimated that 76,250 individuals (44,250 men and 32,000 women) will be diagnosed with and 9,180 men and women will die of melanoma of the skin in 2012 [1]. Increasing incidence is coupled by diagnostic discrepancies [2] whereby a considerable number of clinically suspicious pigmented tumors may show ambiguous histopathology making the classification between benign (common nevus) and malignant (melanoma) melanocytic tumors difficult and the clinical behavior unpredictable [3]. Notwithstanding the distinct sets of genetic alterations demonstrated in melanoma [4], the epigenetic changes remains poorly understood. microRNAs (miRNAs) are endogenous ∼22 nucleotide non-coding small RNAs, which can regulate gene expression in animals and plants by complementary base-pairing to the mRNAs of target genes to specify mRNA cleavage or translation repression [5]. Growing evidence has shown that particular miRNAs function predominately as tumor suppressors, e.g. let-7 family [6], [7] and miR-15a and miR-16 [8]; and some as oncogenes, e.g. miR-17∼92 cluster [9], [10].

The emerging understanding of deregulated expression pattern of miRNAs in many human malignancies has prompted major interest in their discovery and characterization. In fact, a growing number of studies have shown select miRNAs deregulated in melanoma using mostly cell lines [11], [12], [13], [14], [15], [16], [17] and a few using specimens of melanoma metastases [18] or primary tumors [19], [20], [21], [22] based on microarray hybridization or real-time quantitative RT-PCR (qRT-PCR). Although these methods feature reliable clustering of melanomas by similar miRNA expression profiles, they are limited to the detection of known miRNA sequences previously identified by sequencing or homology searches [23]. Alternative Sanger sequencing for miRNA profiling in archived melanoma and nodal specimens [24], while useful in detecting known and novel sequences, were initially complex and expensive due to laborious cloning techniques [25], have now become practical due to the development of next-generation sequencing (NGS).

NGS has proven to be an invaluable tool for the discovery of thousands of miRNA genes because it 1) identifies rare or abundant sequences of known and novel miRNAs; 2) demonstrates extensive sequence variations (isomiRs) [26]; and 3) combines discovery with quantitative expression analysis allowing for digital gene expression profiling [27]. To date, there is no characterization of melanoma miRNA transcriptome based on NGS using a complete set of biopsies representing the steps in melanomagenesis. The current melanoma progression model hypothesizes that through genetic and epigenetic events common nevus (CN) transforms to dysplastic nevus (DN) to melanoma in situ (MIS, non-invasive melanoma, where the melanoma cells are confined to the epidermis) or may become invasive to follow radial and vertical growth phases, culminating in metastatic melanoma [28].

In an effort to gain further insights into the role of miRNAs in melanoma, we have applied the Illumina NGS platform to carry out an in-depth, quantitative comparative analysis of miRNA expression in a discovery cohort fully representing the stages of melanoma progression, i.e. normal skin (NS), CN, thick invasive (T4, AJCC stage [29]) primary cutaneous melanoma (PCM) and metastatic melanomas to lymph node (MMLN, local disease) and to skin (MMS, distant disease). Our results provide a comprehensive view of the miRNA transcriptome in well-defined clinical specimens according to the melanoma progression model showing a repertoire of previously discovered miRNAs while introducing additional known and putative novel candidates exhibiting isomiRs. Furthermore, we reduce the complexity of miRNA transcriptome by defining a set of top-40 miRNAs, which properly classified normal from cancer, validated in a larger independent cohort using qRT-PCR. Finally, we present novel miRNAs, isomiRs and other small RNAs differentially expressed in melanoma that are not yet described.

## Materials and Methods

### Study population and clinical samples

For NGS discovery, the specimens consisted of biopsies or excisions of invasive PCM (n = 5), acrolentiginous melanoma (ALM, n = 1), NS (n = 4), CN (n = 2), MMLN (n = 3), normal lymph node (n = 1) and MMS (n = 3) of patients undergoing curative treatment at Stanford University Medical Center (SUMC) from 1997 onward (Table 1). Some of these specimens, with available clinical follow-up, were matched with the corresponding normal tissues. For qRT-PCR validation, the study cohorts consisted of patients with NS (n = 19), CN (n = 16), DN (n = 19), MIS (n = 17) and PCM (n = 30) undergoing curative treatment at the Dermatology department at University of Connecticut Health Center (UCHC) from 2003 onward (Table S2 in file S1). Two board certified pathologists/deramtopathologists confirmed all rendered diagnoses. For all cohorts, we collected detailed clinicopathologic data on melanomas such as histologic subtype, depth of invasion, ulceration, mitotic index, anatomic level of invasion, tumor infiltrating lymphocytes and regression as described previously [30].

**Table 1:** Clinicopathologic characteristics for discovery cohort.

Sample	Age at diagnosis (years)	Gender	Anatomic site	Breslow's depth, Clark's level	Histological subtype	TNM Staging
***Primary melanoma***						
ALM	61	Male	Sole	3.0 mm, IV	ALM	T3bN1aMx
PCM1^*^	94	Female	Scalp	11.5 mm, V	NM	T4bNxMx
PCM2^#^	52	Male	Scalp	4.0 mm, IV	SSM	T3aN1Mx
PCM3	76	Male	Back	2.8 mm, IV	SSM	T3aN0Mx
PCM4	63	Male	Scalp	2.5 mm, IV	SSM	T3aN0Mx
PCM5	33	Male	Back	2.0 mm, IV	SSM	T2aN1Mx
***Normal skin***						
NS1^*^	94	Female	Scalp			
NS2^#^	52	Male	Scalp			
NS3^ ˆ^	76	Female	Arm			
NS4	40	Male	Knee			
CN1	10	Female	Ear			
CN2	19	Female	Face			
***Metastatic melanoma to lymph node***						
MMLN1	24	Female	Inguinal	(PCM) 1.1 mm, III		T1aN3Mx
MMLN2	NA	NA	NA	NA		TxN1Mx
MMLN3^#^	52	Male	Lateral neck	(PCM) 4.0 mm, IV		T3aN1Mx
***Normal lymph node***						
SLN1^#^	52	Male	Lateral neck			
***Metastatic melanoma to skin***						
MMS1	85	Male	Back	(PCM) 9.5 mm, V		T4aN3M1a
MMS2	NA	NA	NA	NA		TxNxM1a
MMS3^ ˆ^	76	Female	Arm	NA		TxN0M1a

*, #, ˆ denote samples matched with normal control tissues. The abbreviations are: PCM, primary cutaneous melanoma (invasive); ALM, acrolentiginous melanoma; NM, nodular melanoma; SSM, superficial spreading melanoma; NS, normal skin; CN, common nevus; PCM, primary cutaneous melanoma; MMLN, metastatic melanoma to lymph node; MMS, metastatic melanoma to skin; SLN, sentinel lymph node; NA, not available.

Tumor and adjacent normal tissue were collected by surgical resection or biopsy. The great majority of the tissues used for NGS and all those used for qRT-PCR were prepared from deparaffinized FFPE specimens; five tissues were snap-frozen in liquid nitrogen within minutes of collection (Table S1 in file S1). The histology of frozen tissue was examined by the attending pathologist (SSD) for the presence of tumor before RNA extraction. NS was defined as histologically unremarkable skin, 2 cm away from melanoma in excision specimens. The institutional review boards of the Stanford University Medical Center and the University of Connecticut Health Center approved this protocol.

### Sequencing

Total RNA was purified from xylene-extracted FFPE tissue sections with TRIzol (Invitrogen, Carlsbad, CA). Using a nucleotide-specific barcode design [31], we captured the small RNAs by adapter ligation bearing barcode sequence, where multiplex sequencing allowed loading 8 libraries in one lane (Table S1 in file S1). To decrease the chance of sequencing unwanted ribosomal RNAs, ‘poison primers’ were added to the ligated products [24]. After reverse transcription, a cDNA library of mixed barcodes was generated for Illumina genome analyzer (IGA) sequencing. Small RNA libraries were constructed from total RNA as previously described [24] with the following modifications (Figure S1A in file S1): 4 nt barcode tags (Table S1 in file S1) were included in the 5′ adapter oligonucleotide and the libraries were briefly amplified with Illumina specific primers to enable multiplexed sequencing on an Illumina Genome Analyzer (IGA) II. The doubly ligated, purified RNA was reverse-transcribed using 150 U of Superscript II (Invitrogen, Carlsbad, CA) and RT primer, (5′-ATTGATGGTGCCTAC AG-3′). To block the synthesis of contaminating ribosomal RNA, another oligo, 5′-/Bio/GGTG GTATGGCCGTAGAC/InvdT/- 3′(poison primer), was added simultaneously to interfere with the synthesis of the most abundant ribosomal RNA degradation fragment (rGrTrCrTrArCrGrGr CrCrArTrArCrCrArCrC). The resultant cDNA pool was amplified by PCR with the following primers: (forward) 5′-GAT ACG GCG ACC ACC GAG ATC TAC ACT CTT TCC CTA CAC GAC GCT CTT CCG ATC T-3′; and (reverse) 5′- CAA GCA GAA GAC GGC ATA CGA GCT CTT CCG ATC TAT TGA TGG TGC CTA CAG-3′. The ∼125 bp PCR products were purified on a 4% agarose gel followed by gel extraction using a QuiaQuick column (Qiagen, Valencia, CA). To verify the insert-containing libraries prior to NGS, some libraries were cloned for Sanger sequencing into the pCR4-TOPO Cloning vector (Invitrogen, Carlsbad, CA). Following the standard Illumina cluster generation protocol, four uniquely indexed small RNA libraries were pooled, denatured, and loaded on to a single lane of a flow cell for cluster amplification. In the Cluster Station, repeated rounds of polymerase amplification and subsequent denaturation generate clusters of unique sequences on the surface of the flow cell that are then transferred to the Genome Analyzer for sequencing by synthesis. Four fluorescently labeled, reversible terminating nucleotides are successively incorporated then imaged with a high-resolution laser using TIRF (total internal reflection fluorescence) optics to eliminate background and reduce the signal-to-noise ratio. All specimens and cells were multiplexed in 4 separate lanes on the same flow cell; every lane contained a technical replicate, CMELM.

### miRDeep 2.0 analysis

RNA sequencing data in FASTQ form was input into the microRNA-characterization software miRDeep 2.0 on a Linux platform. The software used Bowtie to map the reads to the UCSC reference genome GRCh37 (browser hg19), allowing for only one mismatch and compiling stretches of hairpin sequences up to 140 nt as microRNA precursors. The precursors are then folded into two-dimensional structures using RNAfold from Vienna RNA Package 1.8.5. miRDeep 2.0 evaluated structure and identified miRNAs based on: minimal free energy, length of the 3′ overhang, existing homologues, distribution of passenger (star), guide (mature), loop sequences, and frequency of the predominant read within the sequencing data. A suite of binary scores was applied to each candidate based on experimental nematode and planarian data. The experimental data was used as a basis for generation of Bayesian statistics on each of the relevant features, in short, the natural log of the probability of a known microRNA in nematode and planarian models having a given feature divided by the probability of any background hairpin having that feature. For each particular feature, a candidate in our data then acquired one of two scores, the probability of being a microRNA based on testing positive for the feature, or the probability of being a microRNA based on testing negative for this feature. miRDeep 2.0 incorporates a file of known microRNAs from a curated library at miRBase and bundles corresponding isomirs together, and it generates a ranked list of novel microRNA candidates for follow-on investigation. We checked our final miRNA list against the latest miRBase (v19, released 08/2012) update leading to adaptation of the new nomenclature (3p and 5P) and removal of entities previously recognized as miRNAs and now as others. For example, miR-1274a, miR-1274b, miR-1308, miR-886 and miR-1280, now mostly recognized as tRNAs.

To determine the fold difference for a specific miRNA, we normalized the miRNA sequence count by calculating its percentage per the total sequence count for a given sample. The normalized counts for samples in the same disease were averaged and divided by the average normalized counts for CN or CMELM, melanocytic controls.

### Nearest shrunken centroids (NSC)

To classify diagnostic groups using miRNAs as classifiers, we applied NSC statistical method [32]. miRNA sequence counts determined by miRDeep 2.0 were listed by sample and represented in a tab delimited file as input for an R script (Prediction Analysis for Microarrays or the ‘PAMR’ module). This statistical module analyzed the data based on significance of miRNA expression in any single group. The sequence counts for known miRNAs were normalized using the root-mean-square or the ‘scale’ function in R 2.14.1. The results clustered diagnostic categories and assigned a Z-score statistic that was designed precisely for biomarker detection. The statistics involved the standard error for a particular miRNA added to the standard error for all miRNAs, a ‘positive constant,’ that stabilized the statistics and ensured that miRNAs are not highly ranked simply due to low counts (and thus their low standard error). Instead of settingα-threshold (*P* = 0.05) a priori, we used machine learning to establish ’soft-threshold’ based on a posteriori information learned through the data to best classify specimens based on the smallest number of miRNAs.

The machine-learning method selected parts of the data assigning it to a random ‘test’ bin. Random data were fit to expression values in one of our actual clinically defined diagnostic groups or ‘training sets’. In general, misclassification decreased as more miRNAs were added to the classification process. Based on what was learned through the data, we set an absolute value threshold of 1.85 to our Z-scores. All scores below this absolute value were rounded to 0 and discounted; thereby, we eliminated noisiness and displayed only the most pertinent miRNAs classifying diagnostic groups. A false discovery rate (FDR) was determined by randomly permuting class assignment 100 times, thereby controlling for multiple hypothesis testing. Clustering for all samples was carried out through use of 2*(1-cc) where cc equals a correlation between the cubed root of a value, and the cubed root of a second value or the average of cube-rooted values clustered in a branch by that time point.

Heat map and hierarchical clustering (Euclidian distance, single linkage) were performed with Gene Cluster 3.0 and visualized with Java Treeview. The highest possible correlation between samples was 1 and the lowest is 0, resulting in a dendrogram that presented the clustered samples on a scale of 0 to 2. Higher correlations resulted in shorter distances or heights between samples when branching events occur and tighter groupings.

### Determining RNA biotypes

Sequences from UCSC annotation files wgRNA.txt (snoRNA) and tRNAs.txt (tRNA) and Biomart [33] annotation files on snoRNA, tRNAs, Mt-tRNA, rRNA, snRNA, scRNA, miRNA and lincRNA were compiled into bowtie indexes. These indexes provided us with a library of known RNA molecules and their sites in reference genome GRCh37. Bowtie was used to align sequencing data to the indexes with a norc (no reverse compliment) parameter. Aligned reads that matched to the library were deposited into bins based on RNA type. The quantity of each bin was divided by the total count of aligned sequencing reads to determine the percentage of each RNA type. Z-tests were applied to lincRNA transcripts using SAM-Seq function of the Significance Analysis of Microarrays (SAM) 4.0 software package [34]. Unlike the PAMR module, SAM was more sensitive to low expression values, thus making it the software package of choice for lincRNAs characterized by lower expression.

### Cell lines

Dr. Stanley N. Cohen, Stanford school of medicine, CA, kindly provided established cell lines A2058, A375P, C32, and A375SM, as gifts [35]. We purchased WM983A (Coriell), WM278 (Coriell), WM35 and WM1552C (Wistar institute, Philadelphia, PA). These cells were cultured as previously described [35]. Three types of primary epidermal melanocytesisolated from 3 individuals with light, medium and dark skinwere purchased from ScienCell (Carlsbad, CA) and cultured in melanocyte medium as specified by ScienCell. Cells were incubated at 37°C in a 5% CO2 completely humidified incubator.

### RNA quantification and size

The yield and quality (260/280 O.D. ratios) of RNA were measured by a spectrophotometer (Nanophotometer, Implen, Germany). Small RNA size was measured using Agilent Small RNA Kit with an Agilent 2100 Bioanalyzer (Agilent Technologies, Waldbronn, Germany). The calculated range of captured small RNAs for our library preparation was 144–150 bp; only libraries passing the size criterion were sequenced.

### TaqMan miRNA assay

The expression profile of mature miRNAs for let-7a, let-7b, let-7c, let-7e, let-7f, let-7g and let-7i, miR-211, miR-27b, miR-26b, miR-126, miR-30d, miR-365, miR-150, miR451a and miR451a.1 (custom ordered based on the sequence data) was measured in NS, CN, PCM, MMS, MMLN, and low-passage number cell lines using stem-loop primers for reverse transcription followed by qRT-PCR (TaqMan MicroRNA Assays; Applied Biosystems, Foster City, CA) in a 7500 fast Sequence Detection System (Applied Biosystems). Cycle threshold (Ct) values for each miRNA were normalized vs. small RNA RNU6 (ΔCt) and represented as RQ = 2^−ΔCt^. The expression levels of miRNAs were normalized against U6 non-coding small nuclear RNA. If the U6 were not amplified in a sample, the sample would get flagged and would be excluded from the study. For each sample, 5 ng of total RNA were used for reverse transcription and 1.33 µl of 15 µl reverse transcription product was used for each qRT-PCR. All experiments were carried out in triplicates with appropriate negative control. To determine the fold RQ difference for a specific miRNA among different disease groups, we averaged the RQs from the same disease group divided by average RQ for CN or CMELM.

### Statistical methods

The qRT-PCR data were plotted and analyzed using statistical analysis software SAS version 9.2. To compare miRNA abundance between clinic groups, the data were first logarithmically transformed to achieve normal distribution, which was verified by parallel-notched box-plots. The parallel-notched box-plots not only displayed the basic characteristics of data distribution, including mean, median, IQR (Inter-Quartile Region) and outliers etc., but also allowed for graphic comparisons of miRNA levels between clinical groups. The notches, representing 95% confidence intervals, did not overlap between two groups, which would have significantly different miRNA levels. Statistical significance was tested by comparing logarithmically transformed data among clinic groups using one-way analysis of variance (ANOVA). When the overall test of no group differences from the ANOVA was statistically significant (α = 0.05), *post hoc* pairwise comparisons with Tukey's adjustment were performed to identify group pairs that differed significantly in miRNA abundance (family level of significance α = 0.05). Alternatively, without logarithmic transformation, Kruskal-Wallis test was performed for overall comparisons among clinic groups (α = 0.05). Bonferroni procedure, based on the ranks of the observations, was then used for multiple pairwise comparisons (family level of significance α = 0.10). These methods gave very consistent results. The diagnostic accuracy of each miRNA's abundance was assessed using ROC curves produced in logistic regression models. The sensitivity and (1-Specificity) values were plotted as ROC curves and AUC were calculated using SAS macro codes %ROCPLOT (http://support.sas.com/kb/25/018.html) with minor modification.

### Common Pathway Analysis

We applied a combination of TargetScan, DNA intelligent analysis (DIANA), Kyoto Encyclopedia of Genes and Genomes (KEGG) and miRBase to identify gene targets of our top-40 miRNAs. These miRNAs were statistically significant with a<10% FDR, and were differentially expressed within core diagnostic groups i.e. CN, PCM, MMLN and MMS. Using DIANA mirPath software [36], gene targets were interrogated for miR-144-3p, miR-181b-5p, miR-320a, miR-320c, miR-320d and miR-451a down-regulated in PCM vs. NS libraries; and for miR-203, miR-204-5p (and its homologue, miR-211-5p), miR-205-5p, miR-23b-3p, miR-26a-5p and miR-26b-5p down-regulated in PCM vs. CN libraries. The results consisted of selective KEGG pathways, number of genes, issuing statistical significances assigned to selected pathways based on negative natural logged P-values. Setting the threshold at <0.00095 resulted in combined theoretical gene targets, 514 for PCM vs. NS and 657 for PCM vs. CN interrogation (Table S4 in file S1). Focused on a specific pathway ID, individual KEGG pathway was accessed to pinpoint a putative gene target.

## Results

### Sequencing and annotation of miRNAs

We used two sets of independent cohorts: one for miRNA discovery by NGS and, the other for validation by qRT-PCR. For, discovery, we size-selected (18–30 nt), captured, amplified and sequenced 32 small RNA libraries using an Illumina (Solexa) platform (Figure S1A in file S1). Clinical follow-up and melanoma staging were documented according to the American Joint Committee on Cancer (AJCC) (Table 1). The libraries were prepared from 19 mostly formalin-fixed paraffin-embedded (FFPE) specimens consisting of PCM (mean tumor thickness  = 4.6 mm), matched NS, CN, MMLN and MMS; 9 samples from low-passage number cultured primary melanocytes (CMEL), cultured primary melanoma cell lines (CPM) and cultured metastatic melanoma cell lines (CMM); and 4 technical control replicates of cultured melanocytes of medium skin color (CMELM) in each flow-cell lane (Table S1 in file S1). An unsupervised clustering showed that the miRNA expression levels were nearly identical between the four controls (Figure S1B in file S1), demonstrating reproducibility between lanes. We obtained a total of 4,506,808 small RNA sequences (>17 nt.) with 4,178,297 (86%) sequences mapping to the human genome (hg19) with perfect matches. The average library coverage was 167,467 sequences (range: 25,344–605,910) for individual libraries. The sequenced population averaged 1,351,417 (27.8%) miRNA sequences representing 698 distinct mature known miRNAs. Sequence data is available on gene expression omnibus (GEO, accession number GSE36236).

### Tumor classification and metastatic prediction based on miRNA profiling

To address the quality and stability of cloned small RNAs, we applied strict size criterion using bioanalyzer results. To ensure that the library contained small RNA inserts, only those with single sharp peak (144–150 bp) were sequenced (Figure S2A in file S1); the libraries measuring <130 bp, failing the size criterion were excluded and not sequenced. We compared the quality of small RNA libraries prepared from 4 FFPE primary melanomas to 2 low-passage number primary melanoma cell lines (WM35 and WM278). Both specimen types showed a single sharp peak in the appropriate size range, indicating intact captured small RNAs. Moreover, comparing the small RNA classes for fresh frozen (PCM1) and FFPE (PCM3) melanomas, showed a highly comparable miRNA content, indicating intact miRNAs in FFPE libraries.

**Figure 1:**
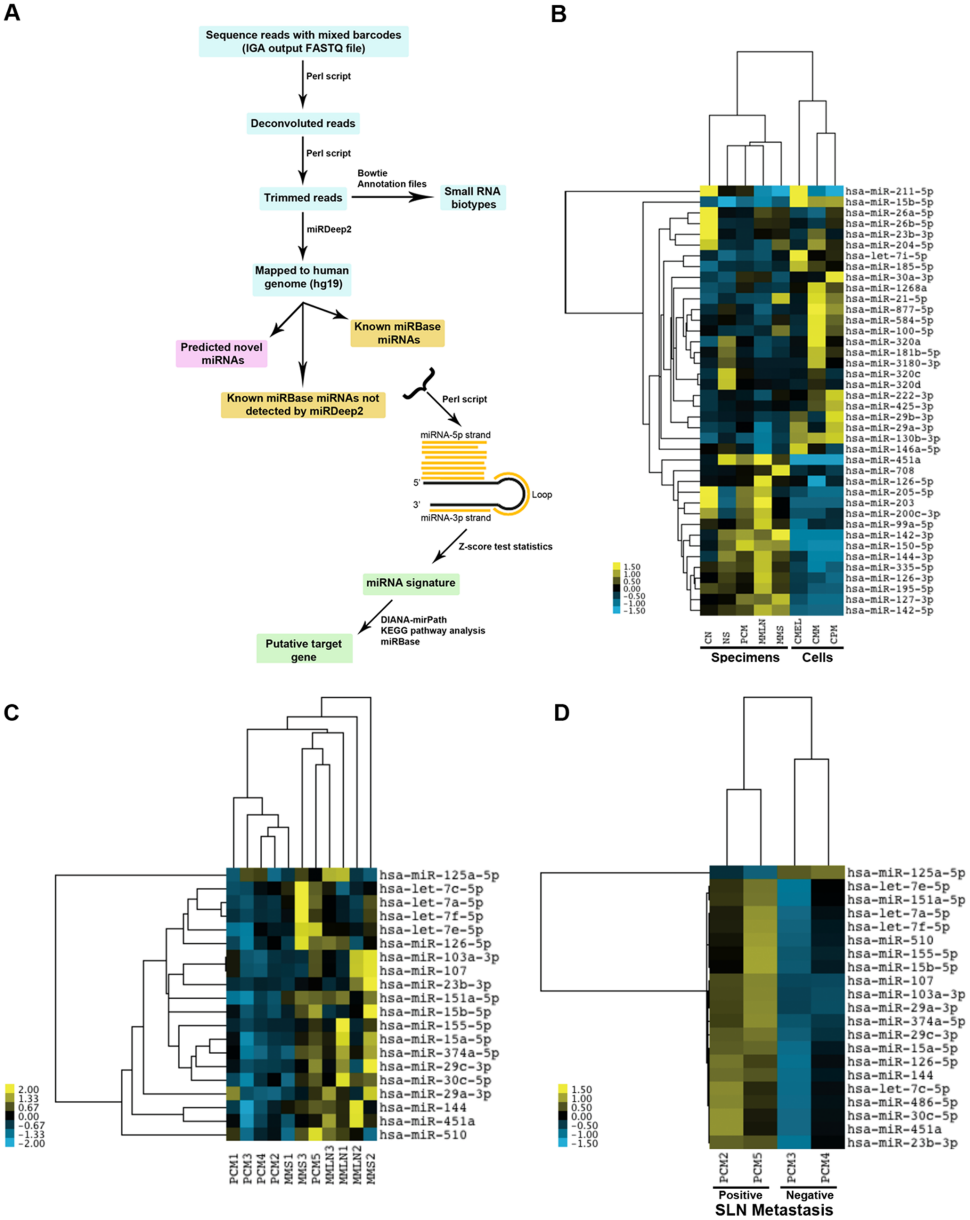
Bioinformatics pipeline, heat map-clustering analysis on top-40 miRNAs differentially expressed in melanoma specimens and cell lines. The flowchart describes the programs and steps used to process the raw small RNA-sequence data to miRNA signature and target gene prediction (A). Clustering analysis of the top-40 miRNAs identified by NGS appropriately segregated primary cutaneous melanoma (PCM), normal skin (NS), common nevus (CN), metastatic melanoma to lymph node (MMLN) and metastatic melanoma to skin (MMS); and cultured primary melanoma (CPM), cultured metastatic melanoma (CMM) and cultured melanocytes (CMEL) (B). The miRNA signature for melanoma specimens is dramatically dissimilar to cell lines. A clustering analysis using 698 distinct mature known miRNAs appropriately segregated PCM from MMLN from MMS (C) and correctly segregated two melanoma patients with biopsy-proven microscopic metastasis to sentinel lymph node (SLN) from two melanoma patients without metastatic disease (D).

Using a series of tools, we processed the raw data showing known miRNAs, predicted novel miRNAs and small RNA classes (Figure 1A). The two main tools were miRDeep 2.0 [37] for assigning miRNA identity; and nearest shrunken centroids (NSC) [32] for statistical analysis. miRDeep 2.0 assigned raw sequences to known miRNAs from miRBase, known miRNAs recognized by miRBase not detected by miRDeep 2.0 and predicted novel miRNAs, providing 1) lists and counts of miRNAs mapping to the mature, the passenger and the loop sequences; 2) possible secondary structures with predicted energy stability; and 3) mapped positions and read counts showing isomiRs. The NSC method classified specimens and cell lines by computing an average miRNA expression vector for each class. These averages were then shrunken towards the overall miRNA expression mean (centroid) across the classes to avoid over-fitting the rank-ordered classifiers that were made up of only a subset of the miRNAs.

After determining miRNA identity, we subjected sequence counts from all libraries to the NSC method and cross-validation (CV), which is a well-established method that repeatedly split samples into training and test sets selecting the optimal number of miRNAs as classifiers in other human cancers [38]. To assess the accuracy of CV, we estimated the false discovery rate (FDR) based on the number of classifiers (Figure S1C in file S1). Choosing the top-40 miRNAs resulted in <10% FDR providing a rank order for miRNAs differentially expressed in all samples (Table 2). To classify benign from malignant lesions, we performed an unsupervised clustering on all the samples showing four major subgroups with an almost-perfect separation between normal and cancer: 1) PCM, CN and NS, 2) MMS and MMLN, 3) CPM and CMM and 4) CMEL (Figure S1D in file S1). For the most part, cases of PCM clustered together separating them from those of NS and CN with the exception of grouping NS1 with CN2, PCM3 with MMLN1-2 and PCM1 with MMLN3. Primary melanoma cell lines (WM35 and C32) were clustered together separate from metastatic cell lines (A375P and A375SM) and melanocytes. Cultured melanocytes of light (CMELL), medium (CMELM) and dark (CMELD) skin pigmentation were segregated from melanoma cell lines and further segregated from each other according to the melanin pigmentation (Figure S1D in file S1). All 4 technical controls tightly clustered together.

**Table 2:** miRNAs discovered in melanoma by NGS compared to other profiling studies.

Rank	miRNA	Other studies
1	hsa-miR-205-5p	[21], [48]
2	hsa-miR-211-5p	[15], [16], [21], [49]
3	hsa-miR-15b-5p	[50]
4	hsa-miR-26a-5p	[51]
5	hsa-miR-451a	
6	hsa-miR-203	[21], [52]
7	hsa-miR-23b-3p	
8	hsa-miR-26b-5p	[11]
9	hsa-miR-877-5p	[43]
10	hsa-let-7i-5p	[50]
11	hsa-miR-142-3p	[17]
12	hsa-miR-30a-3p	[53]
13	hsa-miR-320a	[43]
14	hsa-miR-100-5p	[50]
15	hsa-miR-126-5p	[18]
16	hsa-miR-708	
17	hsa-miR-584-5p	
18	hsa-miR-21-5p	[43], [54], [55]
19	hsa-miR-29b-3p	[51]
20	hsa-miR-1268a	
21	hsa-miR-99a-5p	
22	hsa-miR-150-5p	[18]
23	hsa-miR-146a-5p	[19], [56]
24	hsa-miR-222-3p	[57], [58]
25	hsa-miR-130b-3p	
26	hsa-miR-126-3p	[18]
27	hsa-miR-204-5p	[15], [53]
28	hsa-miR-320c	
29	hsa-miR-200c-3p	[21], [59], [60]
30	hsa-miR-320d	
31	hsa-miR-144-3p	
32	hsa-miR-335-5p	
33	hsa-miR-29a-3p	
34	hsa-miR-127-3p	
35	hsa-miR-181b-5p	
36	hsa-miR-185-5p	[13], [43]
37	hsa-miR-3180-3p	
38	hsa-miR-195-5p	[61]
39	hsa-miR-142-5p	
40	hsa-miR-425-3p	

To verify these results, we assigned miRNA libraries to specific disease groups of NS, CN, PCM, MMLN, MMS, CMEL, CMM and CPM and subjected them to another clustering analysis based on the top-40 list (Table 2). These results classified the disease groups appropriately and segregated PCM from CN, NS, MMS and MMLN (Figure 1B). Similarly, melanoma cell lines – primary melanoma and metastatic – were appropriately separated from melanocytes. Surprisingly, the miRNA signature for melanoma specimens was drastically different from that for melanoma cell lines. In fact, only miR-211 expression was decreased in melanoma specimens (compared to nevi) and cells (compared to melanocytes). Comparing the top-40 list to the published literature on melanoma miRNAs confirmed 23 (58%) previously reported by platforms other than sequencing while introducing an additional 17 (42%) miRNAs (Table 2). Our previous Sanger sequencing identified 7 of the current top-10 miRNAs: miR-205, miR-211, miR-15b, miR-26a, miR-451a, miR-203 and miR-23b [24]. To examine miRNAs associated with metastatic behavior, we subjected PCM, MMLN and MMS to a clustering analysis using 698 distinct mature known miRNAs revealing an almost-perfect separation between primary and melanoma metastases to lymph node and skin (Figure 1C). Using a similar approach with cases of PCM with known sentinel lymph node status, clustering perfectly segregated node-positive patients, those with biopsy-proven microscopic nodal metastasis, from node-negative patients (Figure 1D). Overall, our results showed that the top-40 known miRNAs was sufficient to fairly classify benign from malignant samples.

### Comparison of miRNA profiling by NGS and qRT-PCR

We compared the two platforms, by using qRT-PCR to measure the levels of miR-211, miR451a and miR451a.1 (most abundant isomiR of miR451a), let-7i, miR-203 and miR-205 in two independent cohorts: 1) the very same samples that were sequenced (discovery) and 2) additional, independent specimens (validation, n = 101) (Table S2 in file S1). In the discovery cohort, we compared fold differences between NGS and qRT-PCR for miR-211, let-7i and miR451a sample-by-sample (Figure S3 in file S1). Both platforms showed a significant reduction of miR-211 in melanomas compared to nevi, with NGS showing a higher detection (Figure S2A in file S1). As expected from the heat map results (Figure 1B), no significant changes were detected for let-7i levels among the disease groups by either platform; however, NGS levels of detection were higher than qRT-PCR (Figure S2B in file S1). Both platforms showed a marked reduction of miR-451a (Figure S2C in file S1) and miR-451a.1 (results not shown) levels in PCM compared to NS. Although the overall trends for expression levels were similar between the two platforms, NGS showed a higher detection.

**Figure 2:**
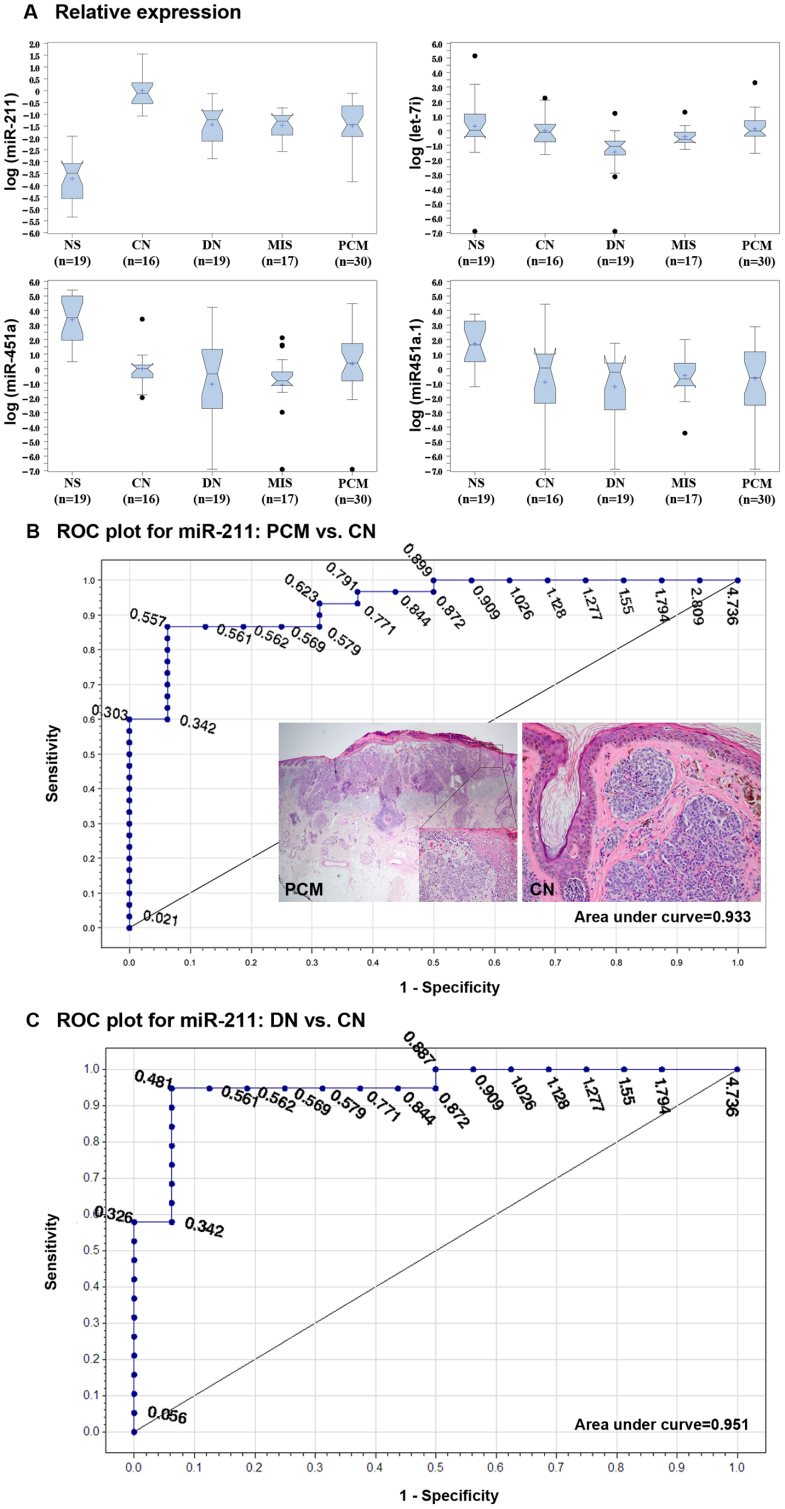
Verification of miRNA expression in validation cohort by qRT-PCR. Relative expression levels of miR-211, let-7i, miR-451a, miR-451a.1, miR-203 and miR-205 (not shown), identified by NGS, were compared between independent patient specimens of normal skin (NS), common nevus (CN), melanoma in situ (MIS) and primary cutaneous melanoma (PCM) (A). Receiver operating characteristic (ROC) curve for miR-211accurately discriminated between diagnostic disease groups, i.e. PCM from CN (AUC = 0.933) (B). The insets show representative PCM and CN used in the study. ROC curve for miR-211accurately discriminated between dysplastic nevus (DN) and CN (AUC = 0.951) (C). The qRT-PCR results were expressed as RQ and shown as log values in boxplots: box starts from 1^st^ quartile and ends at 3^rd^ quartile; cross represents mean; line represents median; notch shows median confidence interval; the ends of whiskers represent the minimum and maximum of the data that are not outliers; and black dots are outliers.

In the validation cohort, we expressed the qRT-PCR results as box plots and analyzed them for statistical significance by Tukey and non-parametric methods (Table S3 in file S1). Both methods showed significantly lower miR-211 levels in DN, MIS and PCM compared to CN; however, no significant difference in miR-211 levels was detected between MIS and PCM (Figure 2A). As previously seen in the discovery cohort, let-7i levels were comparable between NS, CN and PCM; however, let-7i levels were significantly lower in DN compared to CN. The levels for both miR-451a and miR-451a.1 were significantly lower in CN, DN, MIS and PCM than in NS. Although miR-451a levels were lower in MIS compared to CN, it did not reach a statistical significance. The levels for miR-203 and miR-205 were lower in PCM compared to CN, but it did not reach a statistical significance (Table S3 in file S1). Given that the levels of miR-211 were significantly lower in DN, MIS and PCM compared to CN according to disease progression model, we sought if this differential expression could reliably segregate nevi from primary melanomas. Receiver operating characteristic (ROC) curve for miR-211accurately discriminated PCM from CN (Figure 2B, AUC = 0.933) and MIS from CN (AUC = 0.982). Moreover, ROC curve for miR-211 accurately discriminated DN from CN (Figure 2C, AUC = 0.951). The qRT-PCR findings showed 1) overall similar miRNA levels tested in discovery and validation cohorts; and 2) measuring miR-211 levels accurately classified benign and malignant lesions.

### Common pathway analysis

**Figure 3:**
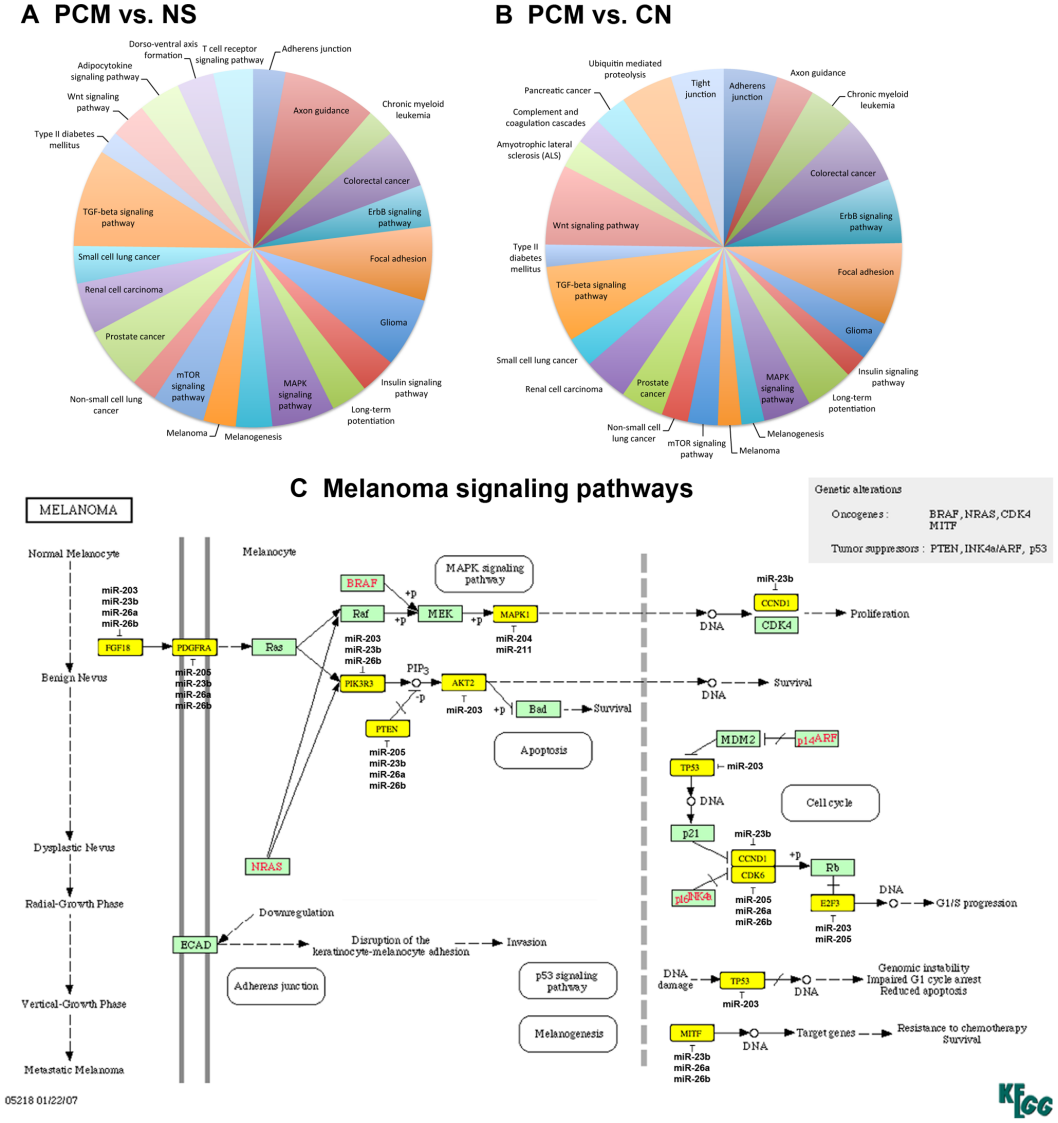
Pathway analysis on deregulated miRNAs in melanoma showed possible combinatorial effects on predicted gene pathways. Interrogating the gene targets for down-regulated miRNAs in PCM vs. NS (A) and in PCM vs. CN (B) showed remarkable similarities in the pathways perturbed. The cutoff *P*-value was set at <0.00095. Common KEGG pathways shared between the two analyses are shown in the same color. Mitogen-activated protein kinase (MAPK) signaling pathway showed that several down-regulated miRNAs could converge on the same putative gene targets (C). The predicted miRNA targets are highlighted in yellow.

To predict the biologic function of our sequence results, we chose to predict targets for co-transcribed miRNAs as seen by clustering together on the y-axis of heat map (Figure 1B). miR-144-3p, miR-181b-5p, miR-320a, miR-320c, miR-320d and miR-451a were down-regulated in PCM vs. NS; and miR-203, miR-211-5p (and its homologue miR-204-5p), miR-205-5p, miR-23b-3p, miR-26a-5p and miR-26b-5p were down-regulated in PCM vs. CN. We interrogated these two series of miRNAs for their gene targets using a combination of TargetScan, DNA intelligent analysis (DIANA), Kyoto Encyclopedia of Genes and Genomes (KEGG) and miRBase. These analyses resulted in a rank-ordered list of KEGG pathways, issuing statistical significance based on negative natural logged *P*-values (Table S4 in file S1). Plotting the predicted KEGG pathways according to down-regulated miRNAs in melanoma vs. normal skin and melanoma vs. nevus showed perturbation in remarkably similar gene pathways (Figure 3A and B). Examining a specific KEGG pathway by down-regulation of miR-203, miR-204-5p, miR-205-5p, miR-211-5p, miR-23b-3p, miR-26a-5p and miR-26b-5p in melanoma highlighted the mitogen-activated protein kinase (MAPK) signaling pathway. Within this pathway, often more than one miRNA is predicted to target FGF18, PDGFRA, PIK3R3, PTEN, AKT2 and MAPK1, where the same gene could be potentially targeted by several co-transcribed miRNAs (Figure 3C). For example, miR-205, miR-23b, miR-26a and miR-26b converge on PDGFRA or miR-211 and miR-204 converge on MAPK1, demonstrating a combinatorial effect of miRNAs on the same target.

### Novel miRNAs and isomiRs in Melanoma

**Figure 4:**
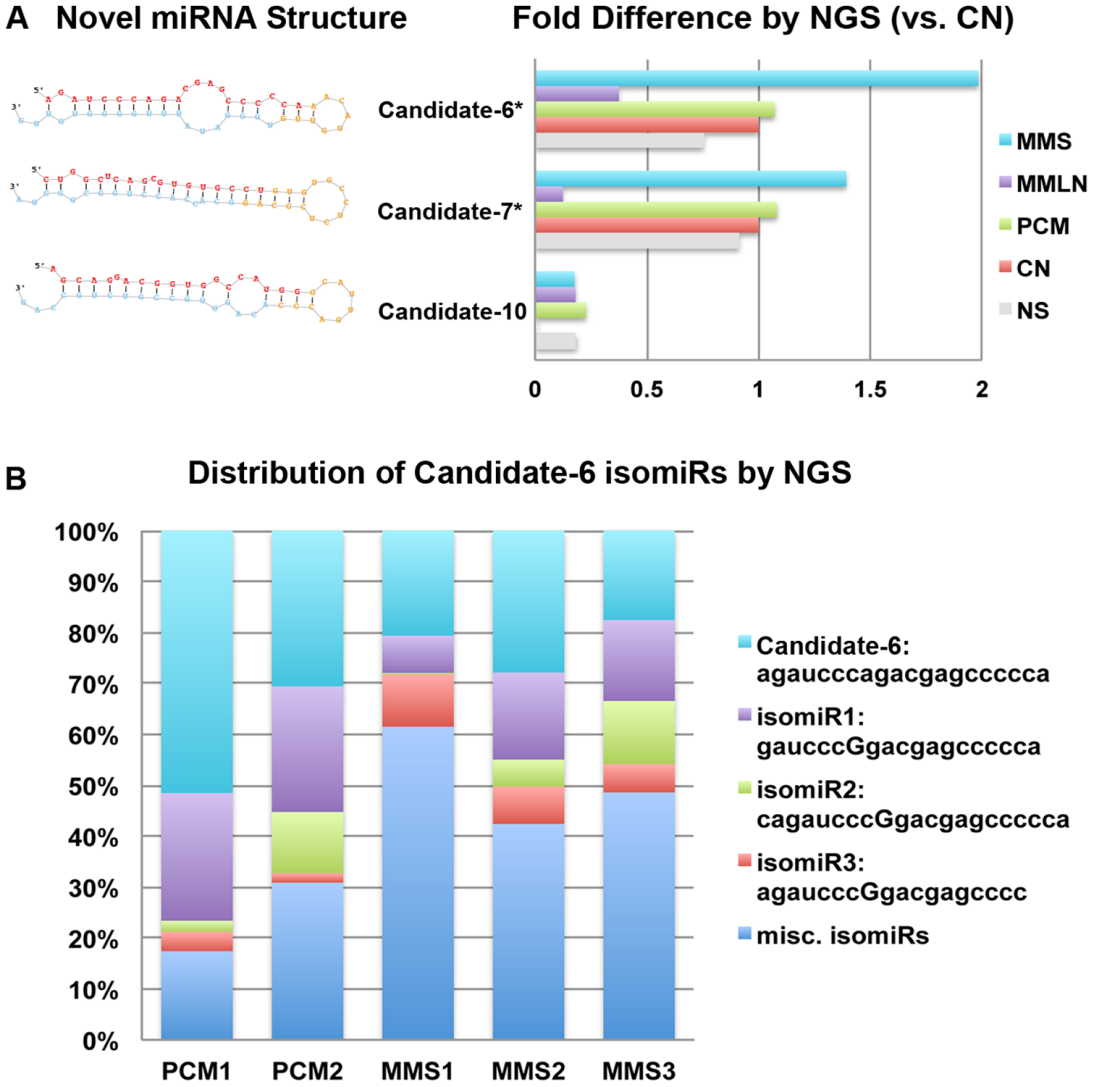
Predicted structures of novel miRNAs, fold differences and isomiR distribution. The putative structures of 3 novel miRNA candidates and their fold differences were compared between disease groups: normal skin (NS), common nevus (CN), primary cutaneous melanoma (PCM), and metastatic melanoma to lymph node (MMLN) and to skin (MMS) (A). The fold difference expression of candidate-6 was significantly higher in MMS compared to PCM (B). Isomeric differences in candidate-6 sequence were compared between PCM and specimens (C).

Using miRDeep 2.0, we identified 429 unique sequences as putative novel miRNAs in specimens based on the following criteria: 1) lowest minimal free energy (MFE), 2) RNA fold forming secondary structures and 3) no similar sequence matches found in miRBase. Using the NSC statistical method and having met Randfold criterion of existing within 5% of the lowest MFE values, we ranked the top-15 putative novel miRNAs out of the 429 sequences. Precursor miRNA sequences with hairpin (50–80 nt) were predicted based on the miRNA-sequence fragments aligning to the 5′ arm, the 3′ arm and the loop sequences (Table S5 in file S1). A BLAT search of hairpin sequences for the top-15 novel miRNAs at the UCSC genome showed chromosomal loci and predicted gene targets (Table S6 in file S1). Of these 15 novel miRNAs, candidates 6, 7 and 10 showed dramatic differences in fold expression when normalized to total miRNA count and compared between disease groups with candidates 6 and 7 showing statistical significance (Figure 4A). Ensemble genome browser revealed that candidate 6 was expressed as a part of SETDB1 transcript. Both candidates 6 and 7 are antisense, suggesting that they are not likely to be degraded fragments of mRNAs. The mapped positions for candidate 6 sequences demonstrated variations in the 5′ (isomiR1) and 3′ (isomiR3) termini and nucleotide substitutions along the miRNA length (isomiRs 1–3) (Figure 4B). To elucidate the extent of isomeric differences in melanoma miRNAs, we examined the read counts of isomiRs for miR-205, miR-211, miR-15b, miR-26a, miR-203, let-7i, miR-142, miR-150, miR-146a and miR-451a (Table S7 in file S1). Surprisingly, this analysis revealed that 6 of 10 most abundant isomiRs were not recognized by miRBase (v18).

### Frequency and classes of small RNAs in melanoma

**Figure 5:**
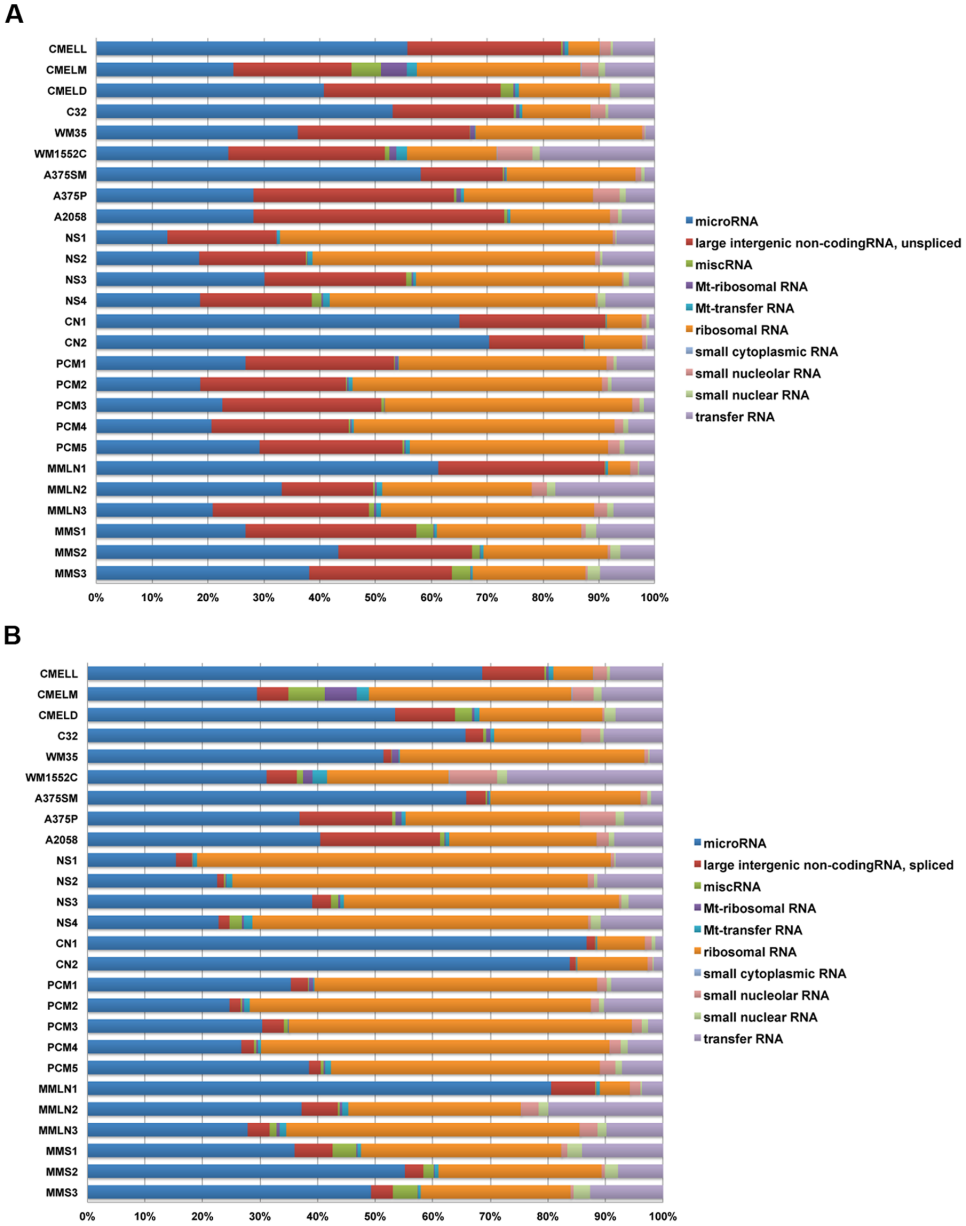
Frequency of small RNA classes in melanoma specimens and cell lines. The stacked bar charts represent an overview of the small RNA abundance (shown in percentages) in all libraries. miRNA, ribosomal RNA and unspliced lincRNA subclasses represented the largest subclasses among the small RNAs. The overall percentage of miRNAs showed an increase when comparing unspliced (A) to spliced lincRNA (B) bar charts.

We subjected libraries to a series of alignment searches to obtain the frequency of non-coding small RNA classes using specific databases for miRNA, large unspliced and spliced intergenic non-coding RNA (lincRNA), miscellaneous RNA (processed, uncategorized transcript), mitochondrial (Mt)-ribosomal RNA, Mt-transfer RNA, ribosomal RNA, small cytoplasmic RNA, small nucleolar RNA, small nuclear RNA and transfer RNA (Figure 5). The three largest classes were derived from miRNA (18–70%), ribosomal RNA (10–59%) and unspliced lincRNA (15–45%) (Figure 5A). Comparing unspliced to spliced lincRNA demonstrated an increase in the miRNA and decrease in the lincRNA (Figure 5B), suggesting that some miRNAs might be spliced products of lincRNAs. Given that lincRNAs can be thousands of bases long, it is surprising that our small RNA sequences (18–30 nt) mapped to lincRNA files. To further investigate this unexpected finding, we compared lincRNAs in PCM to CN libraries looking for statistically significantly altered sequences. One such sequence (exons at chr17: 46,694,460–46,696,607 and 17: 46,699,848–46,699,890) was reduced 87% in PCM compared to CN libraries. Ensembl genome browser localized this sequence to the (-) strand of a 2630 bp non-coding RNA gene (RP11-35H14.16), now a recognized lincRNA (UCSC genome browser ID AK025239), which corresponded to *HOXB8-B9* gene, this locus includes the coding sequence for miR-196a, targeting HOX-C8 mRNA, also down-regulated in melanoma compared to human melanocytes [39]. Another sequence (chr1: 209,605,549–209,605,890) was reduced 73% in PCM libraries compared to CN libraries. This locus (RP11-372M18.2) corresponded to a now recognized lincRNA (UCSC genome browser AK091113) containing miR-205, corroborating with our top ranking miRNA (Table 2).

## Discussion

The two most difficult challenges in managing melanoma patients are lack of practical, robust molecular classification schema and predicting an accurate clinical outcome. Our NGS results show that using miRNAs as classifiers directly from archived biopsies could differentiate benign from malignant, as demonstrated by classifying diverse specimens and cell lines into proper diagnostic groups. Down-regulation of miR-144-3p, miR-181b-5p, miR-320a, miR-320c, miR-320d and miR-451a separated melanoma from normal skin; and down-regulation of miR-203, miR-205, miR-211 (and its homologue, miR-204), miR-23b, miR-26a and miR-26 distinguished melanoma from nevus. The ROC curves for miR-211 discriminated common nevus from melanoma and common nevus from dysplastic nevus in large validation cohort with high specificity and sensitivity, showing the diagnostic utility of this miRNA. Rediscovering miR-211 in our current results served as an important positive control for our NGS results given its anti-invasive role in melanoma cell lines [16] and its intronic location within [15] a known melanoma tumor suppressor gene, *melastatin-1* [40]. Moreover, specific miRNA signature distinguished primary melanomas from metastatic lesions. For example, higher levels of miR-103/107 cluster in primary melanomas were associated with the clinical history of early occult metastasis to sentinel lymph node; similar to a study in breast cancer demonstrating that high level of this miRNA cluster was associated with metastasis and poor outcome [41]. In addition, we found that miR-205 was significantly decreased in primary melanomas and metastases to lymph node compared to nevi. A recent large study of primary melanomas (n = 206) confirmed this down-regulation and showed that it was significantly associated with worse clinical outcome [42].

Although array-based profiling experiments have identified 23 miRNAs deregulated in melanoma thus far, they lack vigor in comprehensively defining the miRNA transcriptome. To date, only one study has deep-sequenced melanoma cell lines [43] and no published study on melanoma biopsies exits. Using melanoma cell lines and clinically defined archived specimens, current study provides an in-depth view of miRNA transcriptome where the previously discovered 23 miRNAs are confirmed while another 17 known sequences are introduced. By combining discovery with digital expression profiling, NGS identified a novel miRNA (candidate 6), which is expressed as a part of SETDB1 transcript, a histone methyltransferase, amplified in melanoma [44] and a candidate gene for melanoma susceptibility [45]. Our NGS results provide a unique look into the isomeric variations of miRNAs deregulated in melanoma specimens and their potential clinical consequence. These so-called isomiRs represent variations of sequences in the 5′ and 3′ termini and nucleotide substitutions along the miRNA length. Microarray-based screening strategies neglect the isomeric differences broadly found in miRNA sequences. For example, we noted that the most abundant read counts of isomiRs for miR-205, miR-211, miR-15b, miR-26a, miR-203, let-7i, miR-142, miR-150, miR-146a and miR-451a, 6/10 top miRNAs deregulated in the specimens, were not represented as the abundant forms in miRBase (v18). Given that most microarrays and commercially available qRT-PCR assays rely on miRBase sequence data, lacking isomeric information could negatively impact discovery of the correct isomiR. This finding may explain the discrepancies between NGS and qRT-PCR platforms in quantitative measurement of a given miRNA levels.

Our previous and current findings demonstrate that miRNAs are stable in FFPE melanoma biopsies and amenable to digital profiling by sequencing, based on the following: 1) the bioanalyzer results showed the same single peak for FFPE melanomas and melanoma lines, the freshest source of total RNA; 2) the miRNA content of frozen melanoma was nearly identical to that of FFPE melanoma; 3) low abundant miRNAs, i.e. 2 copies of miR-203 could be detected by sequencing; 4) the levels of U6 snRNA and miR-211, miR-451a, let-7i, miR-203 and miR-205 were readily amplified and measured using qRT-PCR in 101 archived specimens and 5) having obtained 1,351,417 sequences representing 698 distinct mature known miRNAs. These findings in conjunction with other recent studies [46], [47] establish that miRNA deep sequencing on FFPE cancer tissues is feasible and RNA degradation to the degree observed dose not affect miRNA profiling.

The finding of significant divergence in miRNA-signature between melanoma specimens and low-passage number cell lines further highlight the inherent epigenetic (and genetic) differences between human malignancies and their cultured counterparts, underscoring the use of tumor biopsies directly as the starting material for miRNA discovery in cancer. While cell lines are invaluable for characterizing the cell function of a miRNA or identification of its target(s), they are not ideal starting material for profiling experiments. Generally, most studies have profiled miRNAs from established melanoma cell lines compared to cultured melanocytes by microarray platforms. Using cell models, instead of tumor biopsies, may represent altered cellular and molecular properties as a result of propagation and growth on plastic, making the miRNA results clinically irrelevant. Moreover, a cell line is typically isolated from a metastatic (or primary) lesion in a single patient, not allowing for high throughput profiling of hundreds of tumor biopsies. NGS-based miRNA profiling directly from tumor biopsies is not only disease-specific but also highly informative as microarray platforms are limited to only a few, abundant miRNAs spotted on the array characterized in other cancers. The retained stability of miRNAs in clinical material coupled with NGS platforms could provide a significant advantage to define biomarkers to improve melanoma diagnostics and prognostication.

### Accession number

The IGA sequence data are available in gene expression omnibus (GEO) database (www. Ncbi.nlm.nig.gov/geo/) with accession number GSE36236.

## Supporting Information

File S1 **Contains:** Figure S1. Flow chart for small RNA library construction, sequencing controls, false discovery rate (FDR) and clustering of melanoma specimens and cell lines. Flow chart shows small RNA capturing, amplification and multiplex sequencing (A). Unsupervised clustering of four technical replicates – cultured primary melanocytes from an individual with medium skin color (CMELM) demonstrated a nearly identical miRNA expression pattern between each control (B). Choosing the top-40 miRNAs as classifiers resulted in <10% FDR (C). Unsupervised clustering segregated the primary cutaneous melanoma (PCM) from normal skin (NS), common nevus (CN) and metastatic melanoma to skin (MMS) and lymph node (MMLN) hierarchical clustering of the samples using complete linkage and correlation-based distance (D). Moreover, cultured primary melanoma cell lines (WM35 and C32) clustered together and separated from metastatic cell lines (A375p and A375SM) andmelanocytes. Cultured melanocytes of light (CMELL), medium (CMELM) and dark (CMELD) skin color were segregated according to the melanin content. Figure S2. Comparison of the quality of small RNA library between FFPE primary cutaneous melanomas and primary melanoma cell lines. Both the electrophoresis summary and peak analysis show small RNA library constructed using archived FFPE melanomas (A), s130–s133, and using cell lines (B), WM 35 (EF 35) and WM278 (EF 278). Both FFPE specimens and cell lines showed a single sharp peak in the excepted range 144–150 bp, indicating intact captured small RNAs. Figure S3. Comparison of miRNA expression between NGS and qRT-PCR in discovery cohort. The expression levels of miR-211 (A), let-7i (B) and miR451a (C) were compared between disease groups: normal skin (NS), common nevus (CN), primary cutaneous melanoma (PCM), and metastatic melanoma to lymph node (MMLN) and to skin (MMS). The fold difference by NGS for every miRNA in a given sample was normalized per total miRNA sequence counts for that sample. The fold difference by qRT-PCR was expressed as RQ values for every specimen. Table S1. Illumina (Solexa) flow-cell sample description and barcode sequence used in NGS. Table S2. Clinicopathologic characteristics for validation cohort. Table S3. Pairwise statistical comparisons of miRNA levels to diagnostic groups by Tukey and non-parametric methods. Table S4. Predicted gene pathways and gene targets perturbed by deregulated miRNAs in melanoma. Table S5. Novel miRNA predicted folding, processed and compiled hairpin sequences. Table S6. Novel miRNA chromosomal loci and putative target genes. Table S7. Examples of differences in isomeric read counts of deregulated miRNAs in all specimens combined.
(DOCX)
