# Supplementary material for: In-Depth Characterization of microRNA Transcriptome in Melanoma
Source: PLoS One. 2013 Sep 4;8(9):e72699. doi: 10.1371/journal.pone.0072699 (PMC3762816; doi:10.1371/journal.pone.0072699)
Supplement: File S1 — Contains: Figure S1. Flow chart for small RNA library construction, sequencing controls, false discovery rate (FDR) and clustering of melanoma specimens and cell lines. Flow chart shows small RNA capturing, amplification and multiplex sequencing (A). Unsupervised clustering of four technical replicates – cultured primary melanocytes from an individual with medium skin color (CMELM) demonstrated a nearly identical miRNA expression pattern between each control (B). Choosing the top-40 miRNAs as classifiers resulted in <10% FDR (C). Unsupervised clustering segregated the primary cutaneous melanoma (PCM) from normal skin (NS), common nevus (CN) and metastatic melanoma to skin (MMS) and lymph node (MMLN) hierarchical clustering of the samples using complete linkage and correlation-based distance (D). Moreover, cultured primary melanoma cell lines (WM35 and C32) clustered together and separated from metastatic cell lines (A375p and A375SM) andmelanocytes. Cultured melanocytes of light (CMELL), medium (CMELM) and dark (CMELD) skin color were segregated according to the melanin content. Figure S2. Comparison of the quality of small RNA library between FFPE primary cutaneous melanomas and primary melanoma cell lines. Both the electrophoresis summary and peak analysis show small RNA library constructed using archived FFPE melanomas (A), s130–s133, and using cell lines (B), WM 35 (EF 35) and WM278 (EF 278). Both FFPE specimens and cell lines showed a single sharp peak in the excepted range 144–150 bp, indicating intact captured small RNAs. Figure S3. Comparison of miRNA expression between NGS and qRT-PCR in discovery cohort. The expression levels of miR-211 (A), let-7i (B) and miR451a (C) were compared between disease groups: normal skin (NS), common nevus (CN), primary cutaneous melanoma (PCM), and metastatic melanoma to lymph node (MMLN) and to skin (MMS). The fold difference by NGS for every miRNA in a given sample was normalized per total miRNA sequence counts for that sam [file pone.0072699.s001.docx]

**Supporting Tables**

**Table S1. Illumina (Solexa) flow-cell sample description and barcode sequence used in NGS.**

| **Illumina Lane_Barcode** | **Barcode Sequence** | **Sample** | **Diagnosis** | **Material** |
| --- | --- | --- | --- | --- |
| L5_B1 | Barcode 1: AGCG | MMLN1 | Melanoma metastasis to lymph node | FF |
| L5_B2 | Barcode 2: CGTC | ALM | Acrolentiginous melanoma | FF |
| L5_B3 | Barcode 3: CTGG | MMLN2 | Melanoma metastasis to lymph node | FF |
| L5_B4 | Barcode 4: ACTT | NS1^*^ | Normal skin | FF |
| L5_B5 | Barcode 5: GGGT | PCM1^*^ | Primary cutaneous melanoma | FF |
| L5_B6 | Barcode 6: GTTA | NS2^#^ | Normal skin | FFPE |
| L5_B7 | Barcode 7: TATG | PCM2^#^ | Primary cutaneous melanoma | FFPE |
| L5_B8 | Barcode 8: TCGC | CMELM | Melanocyte, medium skin color | CC |
| L6-B1 | Barcode 1: AGCG | SLN1^#^ | Normal lymph node | FFPE |
| L6-B2 | Barcode 2: CGTC | MMLN3^#^ | Melanoma metastasis to lymph node | FFPE |
| L6-B3 | Barcode 3: CTGG | CN1 | Common nevus | FFPE |
| L6-B4 | Barcode 4: ACTT | CN2 | Common nevus | FFPE |
| L6-B5 | Barcode 5: GGGT | PCM3 | Primary cutaneous melanoma | FFPE |
| L6-B6 | Barcode 6: GTTA | PCM4 | Primary cutaneous melanoma | FFPE |
| L6-B7 | Barcode 7: TATG | PCM5 | Primary cutaneous melanoma | FFPE |
| L6-B8 | Barcode 8: TCGC | CMELM | Melanocyte, medium skin color | CC |
| L7_B1 | Barcode 1: AGCG | CMELD | Melanocyte, dark skin color | CC |
| L7_B2 | Barcode 2: CGTC | MMS1 | Melanoma metastasis to skin | FFPE |
| L7_B3 | Barcode 3: CTGG | MMS2 | Melanoma metastasis to skin | FFPE |
| L7_B4 | Barcode 4: ACTT | MMS3^^^ | Melanoma metastasis to skin | FFPE |
| L7_B5 | Barcode 5: GGGT | NS3^^^ | Normal skin | FFPE |
| L7_B6 | Barcode 6: GTTA | NS4 | Normal skin | FFPE |
| L7_B8 | Barcode 8: TCGC | CMELM | Melanocyte, medium skin color | CC |
| L8_B1 | Barcode 1: AGCG | A2058 | Metastatic melanoma | CC |
| L8_B2 | Barcode 2: CGTC | A375P | Metastatic melanoma | CC |
| L8_B3 | Barcode 3: CTGG | A375SM | Metastatic melanoma | CC |
| L8_B4 | Barcode 4: ACTT | C32 | Primary cutaneous melanoma, amelanotic | CC |
| L8_B5 | Barcode 5: GGGT | WM35 | Primary cutaneous melanoma, RGP | CC |
| L8_B6 | Barcode 6: GTTA | WM1552C | Primary cutaneous melanoma, RGP | CC |
| L8_B7 | Barcode 7: TATG | CMELL | Melanocyte, light skin color | CC |
| L8_B8 | Barcode 8: TCGC | CMELM | Melanocyte, medium skin color | CC |

^*, #, ^^ denote samples matched with normal control tissue. The abbreviations are: FF, fresh frozen; FFPE, formalin-fixed paraffin embedded; CC, cell culture; RGP, radial growth phase; CMELL, cultured melanocytes, light skin color; CMELM, cultured melanocytes, medium skin color; and CMELD, cultured melanocytes, dark skin color. One technical control replicate (underlined) is loaded for every lane.

**Table S2. Clinicopathologic characteristics for validation cohort.**

| **Diagnostic Group** | **NS** | **CN** | **DN** | **MIS** | **PCM** |
| --- | --- | --- | --- | --- | --- |
| **Number of Samples** | 19 | 16 | 19 | 17 | 30 |
| Male | 9 | 9 | 6 | 7 | 21 |
| Female | 10 | 7 | 13 | 10 | 9 |
| **Anatomic Site** |  |  |  |  |  |
| Head & Neck | 3 | 2 | 1 | 3 | 9 |
| Upper Extremity | 2 | 1 | 0 | 4 | 4 |
| Trunk | 13 | 12 | 18 | 7 | 14 |
| Lower extremity | 1 | 1 | 0 | 3 | 3 |
| **Age at diagnosis (years)** |  |  |  |  |  |
| Mean | 58.6 | 36.5 | 43.9 | 53.2 | 54.9 |
| Min | 40 | 10 | 20 | 26 | 18 |
| Max | 94 | 68 | 77 | 79 | 94 |
| **Histology Subtype** |  |  |  |  |  |
| Superficial spreading |  |  |  | 8 | 22 |
| Lentigo maligna |  |  |  | 9 | 4 |
| Nodular |  |  |  | 0 | 3 |
| **Depth of invasion (mm)** |  |  |  |  |  |
| Mean |  |  |  |  | 1.29 |
| Min |  |  |  |  | 0.12 |
| Max |  |  |  |  | 11.5 |
| **Anatomic level of invasion (Clark’s)** |  |  |  |  |  |
| I |  |  |  |  | 1 |
| II |  |  |  |  | 13 |
| III |  |  |  |  | 6 |
| IV |  |  |  |  | 5 |
| V |  |  |  |  | 1 |
| Mean |  |  |  |  | 2.69 |
| **Vertical growth phase** |  |  |  |  |  |
| Absent |  |  |  | 17 | 15 |
| Present |  |  |  | 0 | 14 |
| **Mitoses (/mm^2^)** |  |  |  |  |  |
| 0 |  |  |  |  | 17 |
| 1 |  |  |  |  | 4 |
| 2 |  |  |  |  | 5 |
| 4 |  |  |  |  | 2 |
| 6 |  |  |  |  | 1 |
| **Ulceration** |  |  |  |  |  |
| Absent |  |  |  |  | 29 |
| Present |  |  |  |  | 1 |
| **Tumor Inflammation** |  |  |  |  |  |
| Absent |  |  |  |  | 11 |
| Non-brisk |  |  |  |  | 14 |
| Brisk |  |  |  |  | 5 |
| **Regression** |  |  |  |  |  |
| Absent |  |  |  |  | 24 |
| Present |  |  |  |  | 5 |

NS, normal skin; CN, common nevus; MIS, melanoma in situ; PCM, primary cutaneous melanoma (invasive), IEM, intraepidermal melanocytes.

**Table S3. Pairwise statistical comparisons of miRNA levels to diagnostic groups by Tukey and non-parametric methods.**

|  | **NS** | **CN** | **DN** | **MIS** | **PCM** |
| --- | --- | --- | --- | --- | --- |
| **miR-211** |  | ***** |  |  |  |
|  |  | ***** |  |  |  |
|  |  | ***** |  |  |  |
|  |  | ***** |  |  |  |
|  |  |  | ***** |  |  |
|  |  |  |  | ***** |  |
|  |  |  |  |  | ***** |
|  | | | | | |
| **miR-451a** | ***** |  |  |  |  |
|  | ***** |  |  |  |  |
|  | ***** |  |  |  |  |
|  | ***** |  |  |  |  |
|  | | | | | |
| **miR-451a.1** | ***** |  |  |  |  |
|  | ***** |  |  |  |  |
|  | ***** |  |  |  |  |
|  | ***** |  |  |  |  |
|  | | | | | |
| **Let-7i** | ***** |  |  |  |  |
|  |  | ***** |  |  |  |
|  |  |  |  |  | ***** |
|  | | | | | |
| **miR-203** | **NO SIG** | | | | |
|  | | | | | |
| **miR-205** |  |  | ***** |  |  |

The pairwise comparisons of the two statistically significant disease groups are shown in the same color. *The group with higher miRNA levels. The results for both methods in all disease groups were identical except for miR-205 levels were higher in DN vs. CN by non-parametric method only.

**Table S4. Predicted gene pathways and gene targets perturbed by deregulated miRNAs in melanoma.**

| **PCM vs. NS** | | | | | |
| --- | --- | --- | --- | --- | --- |
| **Down-regulated miRNAs** | **KEGG Pathway** | **Pathway ID** | [**# of Genes (Union)**](http://diana.cslab.ece.ntua.gr/Diana_online_results/pathways/252930221578/kegg_pathways_result_0.php) | [**-ln(p-value) (Union)**](http://diana.cslab.ece.ntua.gr/Diana_online_results/pathways/252930221578/kegg_pathways_result_0.php) | ***P*-value** |
| miR-144-3p | [*TGF-beta signaling pathway*](file://localhost/javascript/view_path('hsa04350_252930221578_0')) | hsa04350 | 28 | 20.02 | 2.02454E-09 |
| miR-181b-5p | [*Axon guidance*](file://localhost/javascript/view_path('hsa04360_252930221578_0')) | hsa04360 | 34 | 19.18 | 4.68916E-09 |
| miR-320a | [*Focal adhesion*](file://localhost/javascript/view_path('hsa04510_252930221578_0')) | hsa04510 | 43 | 15.2 | 2.50847E-07 |
| miR-320c | [*Glioma*](file://localhost/javascript/view_path('hsa05214_252930221578_0')) | hsa05214 | 20 | 14.28 | 6.29387E-07 |
| miR-320d | [*MAPK signaling pathway*](file://localhost/javascript/view_path('hsa04010_252930221578_0')) | hsa04010 | 50 | 12.85 | 2.62963E-06 |
| miR-451a | [*Prostate cancer*](file://localhost/javascript/view_path('hsa05215_252930221578_0')) | hsa05215 | 24 | 12.84 | 2.65605E-06 |
|  | [*Colorectal cancer*](file://localhost/javascript/view_path('hsa05210_252930221578_0')) | hsa05210 | 23 | 12.12 | 5.45628E-06 |
|  | [*mTOR signaling pathway*](file://localhost/javascript/view_path('hsa04150_252930221578_0')) | hsa04150 | 15 | 10.85 | 1.94264E-05 |
|  | [*Renal cell carcinoma*](file://localhost/javascript/view_path('hsa05211_252930221578_0')) | hsa05211 | 19 | 10.5 | 2.75664E-05 |
|  | [Adipocytokine signaling pathway](file://localhost/javascript/view_path('hsa04920_252930221578_0')) | hsa04920 | 18 | 8.58 | 0.000187992 |
|  | [*ErbB signaling pathway*](file://localhost/javascript/view_path('hsa04012_252930221578_0')) | hsa04012 | 21 | 8.55 | 0.000193717 |
|  | [T cell receptor signaling pathway](file://localhost/javascript/view_path('hsa04660_252930221578_0')) | hsa04660 | 21 | 8.09 | 0.000306847 |
|  | [*Small cell lung cancer*](file://localhost/javascript/view_path('hsa05222_252930221578_0')) | hsa05222 | 20 | 7.67 | 0.000466989 |
|  | [Dorso-ventral axis formation](file://localhost/javascript/view_path('hsa04320_252930221578_0')) | hsa04320 | 9 | 7.55 | 0.000526522 |
|  | [*Long-term potentiation*](file://localhost/javascript/view_path('hsa04720_252930221578_0')) | hsa04720 | 16 | 7.47 | 0.00057037 |
|  | [*Insulin signaling pathway*](file://localhost/javascript/view_path('hsa04910_252930221578_0')) | hsa04910 | 28 | 7.47 | 0.00057037 |
|  | [*Melanogenesis*](file://localhost/javascript/view_path('hsa04916_252930221578_0')) | hsa04916 | 22 | 7.44 | 0.000587738 |
|  | [*Wnt signaling pathway*](file://localhost/javascript/view_path('hsa04310_252930221578_0')) | hsa04310 | 29 | 7.38 | 0.000624078 |
|  | [*Melanoma*](file://localhost/javascript/view_path('hsa05218_252930221578_0')) | hsa05218 | 17 | 6.66 | 0.001282031 |
|  | [*Adherens junction*](file://localhost/javascript/view_path('hsa04520_252930221578_0')) | hsa04520 | 17 | 6.66 | 0.001282031 |
|  | [*Chronic myeloid leukemia*](file://localhost/javascript/view_path('hsa05220_252930221578_0')) | hsa05220 | 17 | 5.81 | 0.002999236 |
|  | [*Non-small cell lung cancer*](file://localhost/javascript/view_path('hsa05223_252930221578_0')) | hsa05223 | 13 | 5.21 | 0.005464625 |
|  | [*Type II diabetes mellitus*](file://localhost/javascript/view_path('hsa04930_252930221578_0')) | hsa04930 | 10 | 4.67 | 0.009376809 |
| **PCM vs. CN** | | | | | |
| **Down-regulated miRNAs** | **KEGG Pathway** | **Pathway ID** | [**# of Genes (Union)**](http://diana.cslab.ece.ntua.gr/Diana_online_results/pathways/252930221578/kegg_pathways_result_0.php) | [**-ln(p-value) (Union)**](http://diana.cslab.ece.ntua.gr/Diana_online_results/pathways/252930221578/kegg_pathways_result_0.php) | ***P*-value** |
| miR-203 | [*Focal adhesion*](file://localhost/javascript/view_path('hsa04510_163464721576_0')) | hsa04510 | 54 | 17.29 | 3.10332E-08 |
| miR-204-5p | [*Wnt signaling pathway*](file://localhost/javascript/view_path('hsa04310_163464721576_0')) | hsa04310 | 44 | 16.88 | 4.67594E-08 |
| miR-205-5p | [*TGF-beta signaling pathway*](file://localhost/javascript/view_path('hsa04350_163464721576_0')) | hsa04350 | 31 | 15.99 | 1.13855E-07 |
| miR-211-5p | [*Colorectal cancer*](file://localhost/javascript/view_path('hsa05210_163464721576_0')) | hsa05210 | 29 | 14.31 | 6.10788E-07 |
| miR-23b-3p | [*ErbB signaling pathway*](file://localhost/javascript/view_path('hsa04012_163464721576_0')) | hsa04012 | 29 | 13.56 | 1.29294E-06 |
| miR-26a-5p | [*Adherens junction*](file://localhost/javascript/view_path('hsa04520_163464721576_0')) | hsa04520 | 24 | 11.34 | 1.19018E-05 |
| miR-26b-5p | [Tight junction](file://localhost/javascript/view_path('hsa04530_163464721576_0')) | hsa04530 | 37 | 11.12 | 1.48302E-05 |
|  | [Ubiquitin mediated proteolysis](file://localhost/javascript/view_path('hsa04120_163464721576_0')) | hsa04120 | 36 | 10.95 | 1.7578E-05 |
|  | [*MAPK signaling pathway*](file://localhost/javascript/view_path('hsa04010_163464721576_0')) | hsa04010 | 58 | 10.02 | 4.45472E-05 |
|  | [*Long-term potentiation*](file://localhost/javascript/view_path('hsa04720_163464721576_0')) | hsa04720 | 21 | 10.01 | 4.49949E-05 |
|  | [*Chronic myeloid leukemia*](file://localhost/javascript/view_path('hsa05220_163464721576_0')) | hsa05220 | 24 | 9.96 | 4.73016E-05 |
|  | [*Renal cell carcinoma*](file://localhost/javascript/view_path('hsa05211_163464721576_0')) | hsa05211 | 22 | 9.4 | 8.28047E-05 |
|  | [*Prostate cancer*](file://localhost/javascript/view_path('hsa05215_163464721576_0')) | hsa05215 | 26 | 9.05 | 0.000117501 |
|  | [*Glioma*](file://localhost/javascript/view_path('hsa05214_163464721576_0')) | hsa05214 | 20 | 8.2 | 0.000274887 |
|  | [*Axon guidance*](file://localhost/javascript/view_path('hsa04360_163464721576_0')) | hsa04360 | 32 | 7.99 | 0.000339115 |
|  | [Pancreatic cancer](file://localhost/javascript/view_path('hsa05212_163464721576_0')) | hsa05212 | 21 | 6.92 | 0.000988539 |
|  | [*Small cell lung cancer*](file://localhost/javascript/view_path('hsa05222_163464721576_0')) | hsa05222 | 23 | 6.33 | 0.001783204 |
|  | [*mTOR signaling pathway*](file://localhost/javascript/view_path('hsa04150_163464721576_0')) | hsa04150 | 15 | 6.3 | 0.001837505 |
|  | [Amyotrophic lateral sclerosis (ALS)](file://localhost/javascript/view_path('hsa05030_163464721576_0')) | hsa05030 | 8 | 6.03 | 0.002406998 |
|  | [*Non-small cell lung cancer*](file://localhost/javascript/view_path('hsa05223_163464721576_0')) | hsa05223 | 16 | 5.57 | 0.003812682 |
|  | [Complement and coagulation cascades](file://localhost/javascript/view_path('hsa04610_163464721576_0')) | hsa04610 | 1 | 5.56 | 0.003850996 |
|  | [*Melanoma*](file://localhost/javascript/view_path('hsa05218_163464721576_0')) | hsa05218 | 19 | 4.95 | 0.007087045 |
|  | [*Melanogenesis*](file://localhost/javascript/view_path('hsa04916_163464721576_0')) | hsa04916 | 24 | 4.81 | 0.008151924 |
|  | [*Insulin signaling pathway*](file://localhost/javascript/view_path('hsa04910_163464721576_0')) | hsa04910 | 31 | 4.79 | 0.008316587 |
|  | [*Type II diabetes mellitus*](file://localhost/javascript/view_path('hsa04930_163464721576_0')) | hsa04930 | 12 | 4.66 | 0.009471037 |

The cutoff *P*-value was set at < 0.00095. Common KEGG pathways shared between the two analyses are italicized.

**Table S5. Novel miRNA predicted folding, processed and compiled hairpin sequences.**

| **Candidate** | **MFE** | **Mature (5p)** | **Star (3p)** | **Putative Hairpin^*^** |
| --- | --- | --- | --- | --- |
| 1 | -49.2 | ggcggaggggccgcggg | ugcagccgcuagccug | ggcggaggggccgcgggccggagcucccugcagccgcuagccug |
| 2 | -49.1 | gcugggggcggggagcg | cuuccugcucggcgcgg | cuuccugcucggcgcggacgguaggagcccucggaggaggcauccuucauaacgcugggggcggggagcg |
| 3 | -58.8 | cggggagcgcggcgggc | ccgccuugccuccuuccgg | ccgccuugccuccuuccgggccucucgggccccggggagcgcggcgggc |
| 4 | -38.9 | ccccggacaagccccca | gggcucuagucccacaggca | gggcucuagucccacaggcaaaggagacaguaaaucugucaucuucaaccccggacaagccccca |
| 5 | -54.7 | cgccggggcgcgccggc | ccggcgcccccgccccg | cgccggggcgcgccggccugcgcccaacggucaccgccgcccccaccugggccgcaaccgccggcgcccccgccccg |
| 6 | -35.5 | agaucccagacgagccccca | ugggauauuugggguguug | agaucccagacgagcccccaaacauguugugggauauuugggguguug |
| 7 | -45.2 | uggcucagcgugugccu | gcaccaccuggcgggg | uggcucagcgugugccugugugccucucgcaggcaccaccuggcgggg |
| 8 | -41.8 | ccucacacggggcacca | ggcuccaacugugggggg | ggcuccaacuguggggggucuguuuccgcagaccgcugacuuuccucacacggggcacca |
| 9 | -63.2 | cgcgggugggggggcggg | cgacuccugcccgggcg | cgcgggugggggggcggggcaggcuggccgcgugcgugccgacuccugcccgggcg |
| 10 | -36.5 | agcaggacgguggccau | aggugccgucugccag | uggcucagcgugugccugugugccucucgcaggcaccaccuggcgggg |
| 11 | -50.5 | agcgaggggggcccggg | cgggccauuucgcaaa | agcgaggggggcccgggggcugacccccagcgacccgcgggccauuucgcaaa |
| 12 | -47.2 | ggcgggugcggggguggg | uagccccugcugaaugaag | uagccccugcugaaugaaggagaggcgggugcggggguggg |
| 13 | -34.3 | uuccaguuuucccaggaaa | uccuggaaugugggacuu | uuccaguuuucccaggaaagagagguuccuggaaugugggacuu |
| 14 | -21.3 | gucuacggccauaccacc | ggucuacggccguaccacc | ggucuacggccguaccacccuaaaugcacccgaucucaucaaaauaaggcagcagucuacggccauaccacc |
| 15 | -36.7 | ugagguaggagguuguga | aagacccaugcaugccccagu | ugagguaggagguugugaaacucugguggcaccuaggaccuaggaaugccuuuuuaagaggcaagacccaugcaugccccagu |

^*^The actual size of the stem-loop has not been experimentally determined.

**Table S6. Novel miRNA chromosomal loci and putative target genes.**

| **Candidate** | **Chr** | **Loci Start** | **Loci End** | **Strand** | **Gene Target** | **Nested** | **Homologue** |
| --- | --- | --- | --- | --- | --- | --- | --- |
| 1 | 1 | 234040741 | 234040784 | (-) | SLC35F3 (+) | No | No |
| 2 | 4 | 10458445 | 10458514 | (+) | ZNF518B (-) | No | hsa-miR-762 |
| 3 | 5 | 175665743 | 175665791 | (+) | C5orf25 (+) | Intron | hsa-miR-4532 |
| 4 | 7 | 50110114 | 501101178 | (+) | ZPBP (-) | No | No |
| 5 | 10 | 21823751 | 21823827 | (+) | MLLT10 (+) | Intron | No |
| 6 | 11 | 386805 | 386852 | (-) | SETDB1 (+) | No | No |
| 7 | 12 | 5950238 | 5950287 | (-) | ANO2 (-) | Intron | hsa-miR-4322 |
| 8 | 12 | 95841364 | 95841423 | (+) | Noncoding | No | No |
| 9 | 14 | 23449991 | 23450046 | (-) | JUB (-) | Intron | hsa-miR-3656 |
| 10 | 16 | 72089402 | 72089448 | (-) | TNXL4B (-) | Intron | No |
| 11 | 17 | 2303166 | 2303218 | (+) | MNT (-) | No | No |
| 12 | 19 | 51924892 | 51924932 | (-) | Noncoding | No | hsa-miR-3656, hsa-miR-4763 |
| 13 | 21 | 23880933 | 26880976 | (-) | Noncoding | Intragenic region, found near active regulatory element H3K27Ac | hsa-miR-145, has-miR-4328 |
| 14 | 22 | 36713014 | 36713085 | (+) | MYH9 (-) | No | No |
| 15 | X | 90284199 | 90284281 | (+) | Noncoding | No | hsa-let-7a, hsa-let-7b, hsa-let7c,  hsa-let-7e |

**Table S7. Examples of differences in isomeric read counts of deregulated miRNAs in all specimens combined.**

| **miRNA isomiRs** | **Length** | **Counts** | **miRBase (v18) name and sequence** | **miRBase accession number** |
| --- | --- | --- | --- | --- |
| miR-205 |  |  | >hsa-miR-205-5p | MIMAT0000266 |
| uccuucauuccaccggagucugu | 23 | 129 |  |  |
| **uccuucauuccaccggagucug** | 22 | 5058 | uccuucauuccaccggagucug |  |
| uccuucauuccaccggagucu | 21 | 21396 |  |  |
| uccuucauuccaccggaguc | 20 | 1210 |  |  |
| uccuucauuccaccggagu | 19 | 2377 |  |  |
| uccuucauuccaccggag | 18 | 101 |  |  |
|  |  |  |  |  |
| miR-211 |  |  | >hsa-miR-211-5p | MIMAT0000268 |
| uucccuuugucauccuucgccua | 23 | 55 |  |  |
| **uucccuuugucauccuucgccu** | 22 | 3836 | uucccuuugucauccuucgccu |  |
| uucccuuugucauccuucgcc | 21 | 626 |  |  |
| uucccuuugucauccuucgc | 20 | 294 |  |  |
| uucccuuugucauccuucg | 19 | 62 |  |  |
|  |  |  |  |  |
| miR-15b |  |  | >hsa-miR-15b-5p | MIMAT0000417 |
| **uagcagcacaucaugguuuaca** | 22 | 2799 | uagcagcacaucaugguuuaca |  |
| uagcagcacaucaugguuuac | 21 | 5584 |  |  |
| agcagcacaucaugguuuac | 20 | 32 |  |  |
| uagcagcacaucaugguuua | 20 | 1737 |  |  |
| uagcagcacaucaugguuu | 19 | 759 |  |  |
| cagcacaucaugguuuaca | 19 | 32 |  |  |
| uagcagcacaucaugguu | 18 | 121 |  |  |
| uagcagcacaucauggu | 17 | 35 |  |  |
|  |  |  |  |  |
| miR-26a |  |  | >hsa-miR-26a-5p | MIMAT0000082 |
| uucaaguaauccaggauaggcug | 23 | 136 |  |  |
| **uucaaguaauccaggauaggcu** | 22 | 44235 | uucaaguaauccaggauaggcu |  |
| uucaaguaauccaggauaggc | 21 | 32871 |  |  |
| uucaaguaauccaggauagg | 20 | 21015 |  |  |
| uucaaguaauccaggauag | 19 | 4993 |  |  |
| uucaaguaauccaggaua | 18 | 1108 |  |  |
| uucaaguaauccaggau | 17 | 1635 |  |  |
|  |  |  |  |  |
| miR-203 |  |  | >hsa-miR-203 | MIMAT0000264 |
| ugaaauguuuaggaccacuaga | 22 | 8 |  |  |
| **gugaaauguuuaggaccacuag** | 22 | 47 | gugaaauguuuaggaccacuag |  |
| gugaaauguuuaggaccacua | 21 | 28 |  |  |
| ugaaauguuuaggaccacuag | 21 | 266 |  |  |
| gugaaauguuuaggaccacu | 20 | 21 |  |  |
| ugaaauguuuaggaccacua | 20 | 5 |  |  |
| gugaaauguuuaggaccac | 19 | 8 |  |  |
| ugaaauguuuaggaccacu | 19 | 2 |  |  |
| ugaaauguuuaggacca | 17 | 2 |  |  |
|  |  |  |  |  |
| let-7i |  |  | >hsa-let-7i-5p | MIMAT0000415 |
| ugagguaguaguuugugcuguug | 23 | 7 |  |  |
| **ugagguaguaguuugugcuguu** | 22 | 4716 | ugagguaguaguuugugcuguu |  |
| ugagguaguaguuugugcugu | 21 | 2193 |  |  |
| gagguaguaguuugugcuguu | 21 | 46 |  |  |
| ugagguaguaguuugugcug | 20 | 927 |  |  |
| ugagguaguaguuugugcu | 19 | 1585 |  |  |
| gguaguaguuugugcuguu | 19 | 49 |  |  |
| guaguaguuugugcuguu | 18 | 28 |  |  |
| ugagguaguaguuugugc | 18 | 976 |  |  |
| ugagguaguaguuugug | 17 | 859 |  |  |
| uaguaguuugugcuguu | 17 | 119 |  |  |
|  |  |  |  |  |
| miR-142-3p |  |  | >hsa-miR-142-3p | MIMAT0000434 |
| guaguguuuccuacuuuaugga | 22 | 933 |  |  |
| **uaguguuuccuacuuuaugga** | 21 | 78 | uguaguguuuccuacuuuaugga |  |
| uguaguguuuccuacuuuaug | 21 | 111 |  |  |
| guaguguuuccuacuuuaugg | 21 | 110 |  |  |
| uguaguguuuccuacuuuau | 20 | 153 |  |  |
| guaguguuuccuacuuuaug | 20 | 81 |  |  |
| uguaguguuuccuacuuua | 19 | 51 |  |  |
| guaguguuuccuacuuuau | 19 | 52 |  |  |
| uguaguguuuccuacuuu | 18 | 68 |  |  |
|  |  |  |  |  |
| miR-150 |  |  | >hsa-miR-150-5p | MIMAT0000451 |
| ucucccaacccuuguaccagugc | 23 | 64 |  |  |
| **ucucccaacccuuguaccagug** | 22 | 1350 | ucucccaacccuuguaccagug |  |
| ucucccaacccuuguaccagu | 21 | 1204 |  |  |
| ucucccaacccuuguaccag | 20 | 233 |  |  |
| ucucccaacccuuguacca | 19 | 54 |  |  |
|  |  |  |  |  |
| miR-146a |  |  | >hsa-miR-146a-5p | MIMAT0000449 |
| ugagaacugaauuccauggguug | 23 | 76 |  |  |
| **ugagaacugaauuccauggguu** | 22 | 885 | ugagaacugaauuccauggguu |  |
| gagaacugaauuccauggguu | 21 | 33 |  |  |
| ugagaacugaauuccaugggu | 21 | 946 |  |  |
| ugagaacugaauuccauggg | 20 | 161 |  |  |
| ugagaacugaauuccaugg | 19 | 123 |  |  |
| aacugaauuccauggguu | 18 | 309 |  |  |
| acugaauuccauggguu | 17 | 45 |  |  |

The underlined denote counts for the most abundant isomiR. The boldface denotes perfect sequence match to miRBase (v18).

**
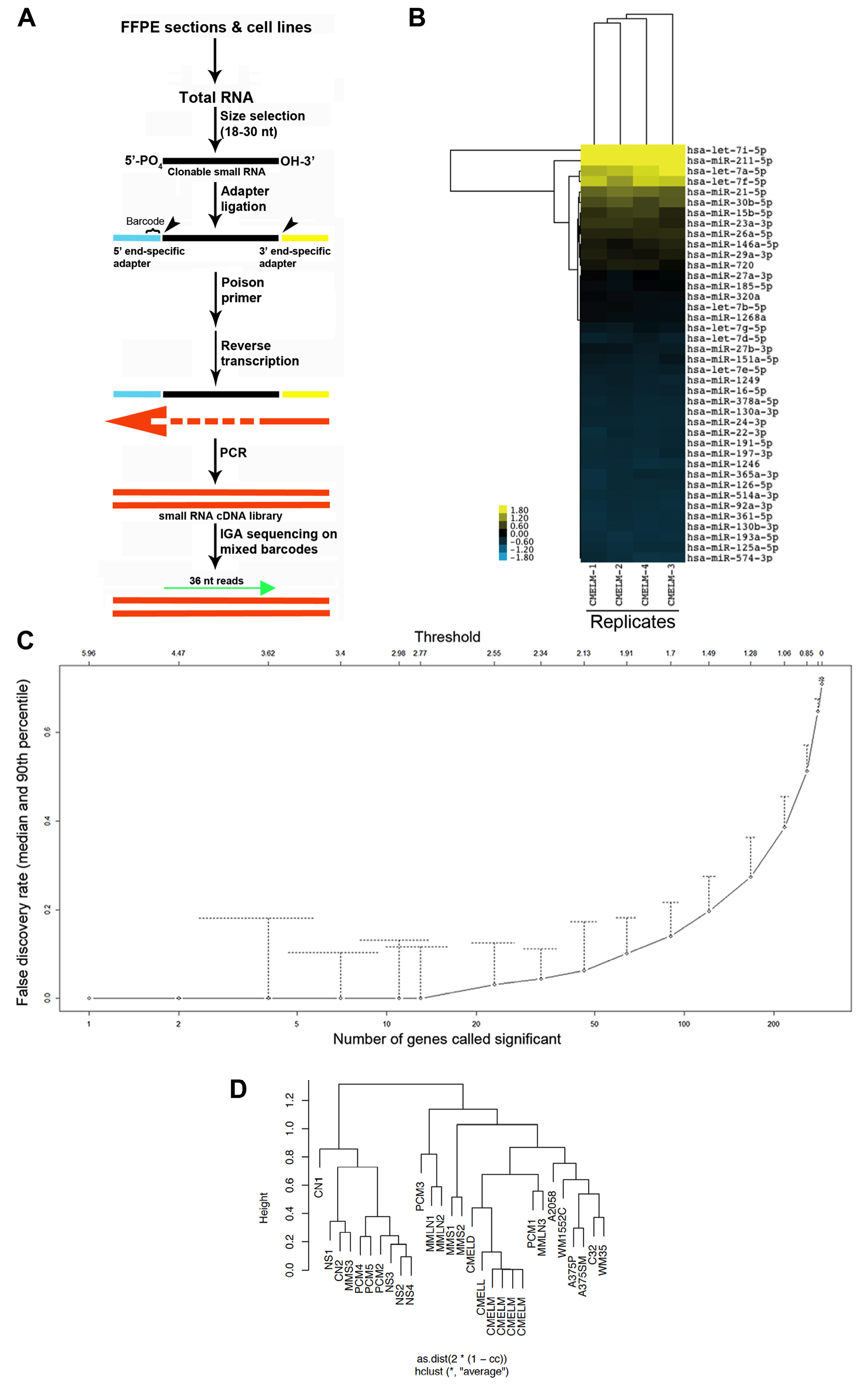
**

**Figure S1**

**
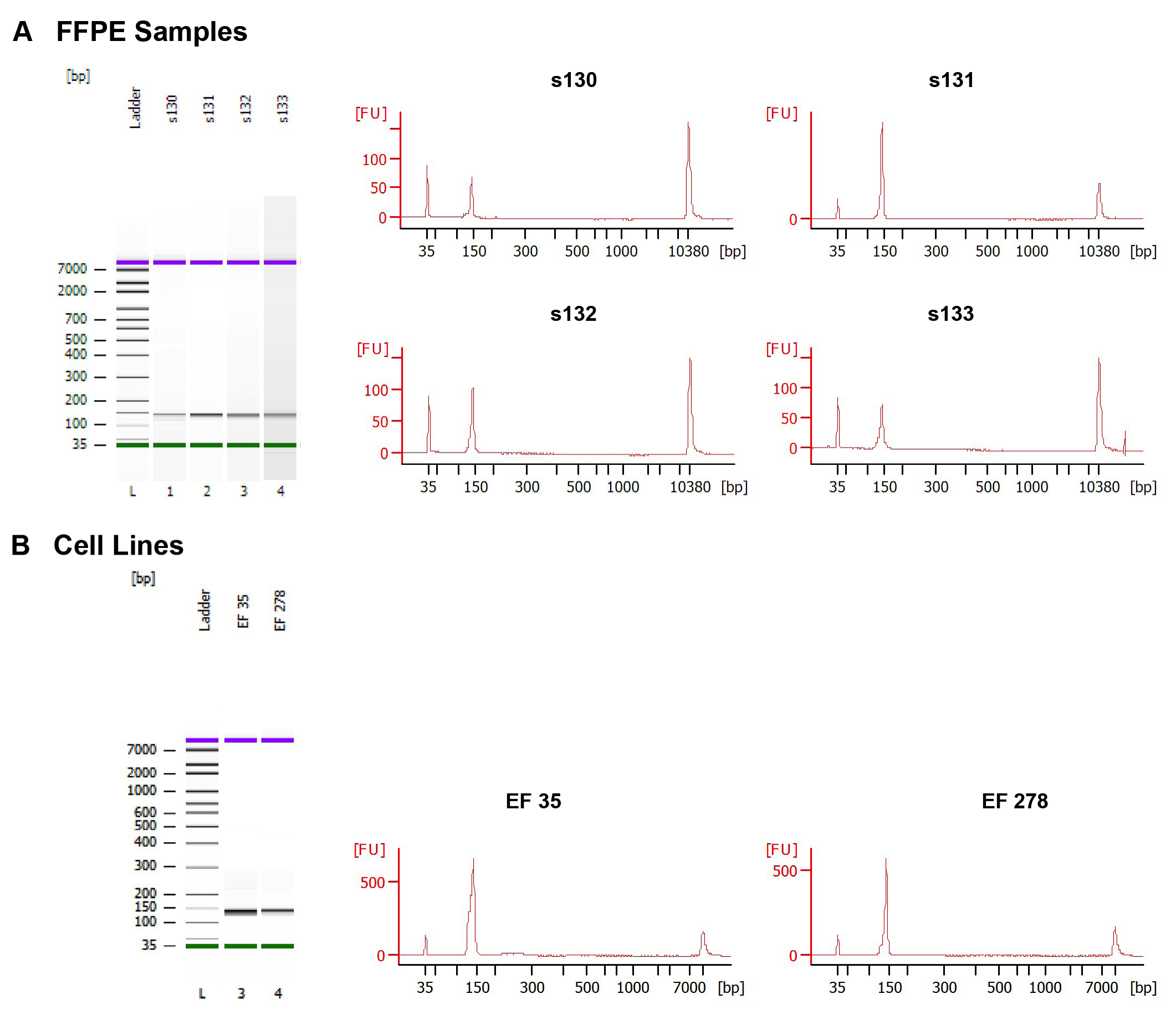
**

**Figure S2**

**
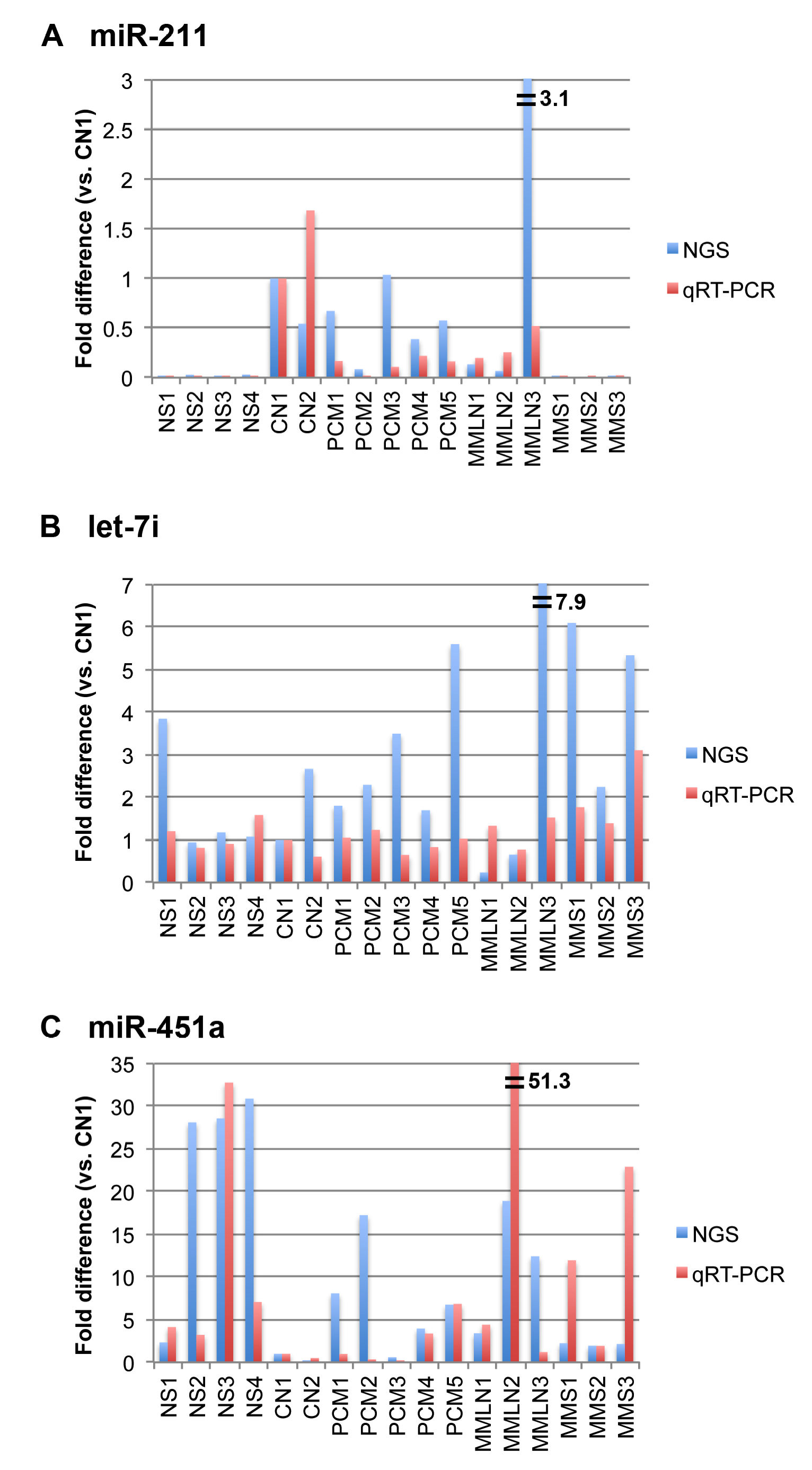
**

**Figure S3**
